# State-of-the-Art of Myocardial Perfusion by CMR: A Practical View

**DOI:** 10.31083/j.rcm2310325

**Published:** 2022-09-26

**Authors:** Guillem Pons-Lladó, Peter Kellman

**Affiliations:** ^1^Head (Emeritus), Cardiac Imaging Unit, Cardiology Department, Hospital de Sant Pau, Universitat Autònoma de Barcelona, Clínica Creu Banca, 08034 Barcelona, Spain; ^2^National Heart, Lung, and Blood Institute, National Institutes of Health, Department of Health and Human Services, Bethesda, MD 20892, USA

**Keywords:** perfusion magnetic resonance imaging, ischemic heart disease, computational intelligence

## Abstract

Ischemic heart disease (IHD) outstands among diseases threatening public health. Essential for its management are the continuous advances in medical and interventional therapies, although a prompt and accurate diagnosis and prognostic stratification are equally important. Besides information on the anatomy of coronary arteries, well covered nowadays by invasive and non-invasive angiographic techniques, there are also other components of the disease with clinical impact, as the presence of myocardial necrosis, the extent of pump function impairment, and the presence and extent of inducible myocardial ischemia, that must be considered in every patient. Cardiovascular Magnetic Resonance (CMR) is a multiparametric diagnostic imaging technique that provides reliable information on these issues. Regarding the detection and grading of inducible ischemia in particular, the technique has been widely adopted in the form of myocardial perfusion sequences under vasodilator stress, which is the subject of this review. While the analysis of images is conventionally performed by visual inspection of dynamic first-pass studies, with the inherent dependency on the operator capability, the recent introduction of a reliable application of quantitative perfusion (QP) represents a significant advance in the field. QP is based on a dual-sequence strategy for conversion of signal intensities into contrast agent concentration units and includes a full automatization of processes such as myocardial blood flow (MBF) calculation (in mL/min/g), generation of a pixel-wise flow mapping, myocardial segmentation, based on machine learning, and allocation of MBF values to myocardial segments. The acquisition of this protocol during induced vasodilation and at rest gives values of stress/rest MBF (in mL/min/g) and myocardial perfusion reserve (MPR), both global and per segment. Dual-sequence QP has been successfully validated against different reference methods, and its prognostic value has been shown in large longitudinal studies. The fact of the whole process being automated, without operator interaction, permits to conceive new interesting scenarios of integration of CMR into systems of entirely automated diagnostic workflow in patients with IHD.

## 1. Introduction

Ischemic heart disease (IHD) has several components that conditionate its impact in terms of physical disability, likelihood of future events, and, ultimately, death of the sufferers. These factors can be summarized as: (1) extent and severity of coronary artery atherosclerotic lesions; (2) presence and extent of infarcted myocardium; (3) degree of contractile dysfunction; and (4) extent and significance of inducible ischemia. Therapies for the disease are well established but eventually tailored to every patient in clinical practice, depending on the relative weight of each one of these elements. Essential for the efficiency of this process of management of IHD is thus an accurate and reliable clinical assessment of their particular role in every stage of the disease.

Diagnostic imaging techniques have been in use in cardiology for the last 50 years, but the process of clinical acceptance of those most recently introduced, as Cardiovascular Magnetic Resonance (CMR) and Computed Tomography (CCT), has been particularly fast, after an extensive demonstration of their value since the early 2000s. Nowadays, thus, it is accepted that a rational use of CMR and CCT may provide a comprehensive body of information on the aforementioned components of IHD, which is supported, not only by recommendations issued by committees of experts on each technique [1, 2, 3], but also by current official guidelines endorsed by scientific societies of cardiology [4].

There is little doubt that CCT has largely accomplished the expectations it aroused when it was introduced [5]. Its ability for providing the clinician with non-invasive, reliable information on the anatomy of the coronary vessels, which is the cornerstone of the diagnosis of coronary artery disease (CAD), has led cardiologists to prescribe increasingly the technique, where available, as a first line test in patients with symptoms consistent with the disease [6]. Once CAD has been diagnosed, or when chronic IHD is already known to be present, clinicians have a good deal of diagnostic methods at hand to complement the study of patients, but CMR stands out as an excellent alternative [1]. Thus, on one hand, the accurate information it provides on myocardial function and tissue composition (i.e., necrotic scar) is essential for establishing a baseline profile of every patient with IHD, which is helpful for risk stratification. On the other hand, CMR methods testing the presence of inducible ischemia constitute an important element in decision making for the most appropriate treatment in each case. The present article will review the state-of-the-art of CMR myocardial perfusion under stress agents for the assessment of this important subject in current clinical practice.

## 2. Myocardial Ischemia: Evolving Concepts and Study Methods 

In a strict sense, myocardial ischemia occurs at any time when oxygen supply does not meet tissue metabolic demands, the most prevalent underlying cause being impaired perfusion due to CAD [7]. If an ischemic episode is long enough, a series of events are elicited which develop consecutively within minutes, constituting the so-called “ischemic cascade” [8]: thus, once regional hypoperfusion is established, myocardial dysfunction appears, first diastolic and, then, systolic, followed by ECG changes and, eventually, by the perception of anginal pain. Obviously, the maintenance of a severely reduced or abolished flow over time leads to acute myocardial infarction (MI), while a chronic state of reduced myocardial perfusion severe enough may derive into hibernating myocardium.

As the diagnosis of ischemia by direct measurement of myocardial tissue oxygenation is not feasible in clinical practice, investigators focused historically on studying aspects potentially related with, either, its cause (i.e., severity of coronary artery stenoses), or its consequences (i.e., observation of stress-induced changes from the ischemic cascade). This led to the acquisition of several concepts that are still widely in use, as is the significance of a reduction in >70% of the coronary lumen at angiography as a source of ischemia. This threshold was chosen as the one where the curve of increased regional myocardial flow under induced hyperemia showed a particularly steep drop, in animal experimentation [9]. The concept of coronary flow reserve (CFR) was thus coined as a referential physiological indicator of impeded flow. Alternatively, attention was put on the potential as surrogates of the ischemic process of the components of the ischemic cascade when induced by some form of stress. On these grounds, non-invasive procedures able to show the appearance of ECG-changes [10] or contractile dysfunction [11] found their place and are also routine in current practice nowadays.

Advances in both hemodynamic and non-invasive media have contributed to introduce new concepts and helped to refine the diagnosis of myocardial ischemia. On the invasive side, after warning calls upon the risk of considering exclusively angiographic measures of vessel stenoses as an indicator of significant obstruction [12], physiological invasive measurement procedures have been developed. The most relevant in practice have been the method of Fractional Flow Reserve (FFR), that estimates the pressure drop across an obstructive coronary lesion under maximal vasodilatation, and the Instantaneous free-wave Ratio (iFR), a simplified technique that does not require induced hyperemia [13]. These techniques are specific, and an invasive standard of reference, for the study of discrete epicardial stenoses, in contrast with the Index of Microcirculatory Resistance (IMR), a new thermodilution-based method for the assessment of coronary microvasculature [14], still not widely available in current practice.

The approach of radionuclide studies to the field is based on the direct relationship between myocardial blood flow (MBF) and radiotracer uptake by myocardial cells. The resulting ECG-gated Single-Photon-Emission-Computed-Tomography (SPECT) presents with 3D maps of relative myocardial perfusion allowing for the detection of potential defects when comparing stress and rest acquisitions [15], this being probably the most commonly used method of perfusion imaging nowadays. Particularly relevant in the study of myocardial perfusion is the technique that uses positron-emitting radiotracers and dedicated PET scanners: the metabolic shift in the ischemic myocardium from free-fatty acids to glucose [16] can be detected by the incorporation into this pathway of glucose-analog positron-emitting tracers, this allowing for the technique to be considered a truly metabolic one [17], which is theoretically the closest we may get to the very concept of ischemia stated above. In addition, and most important, the use of PET perfusion tracers with appropriate rates of blood extraction and myocardial retention and, thus, a nearly lineal relationship between MBF and the measured tracer activity, permits an absolute quantification of regional MBF, in terms of volume of flow per time and left ventricular mass units (mL/min/g) [18], which is the formal expression of myocardial perfusion. Although not widely used in current clinical practice for logistical reasons, Rest and Stress MBF and the derived Myocardial Perfusion Reserve (MPR) by PET are the standard of reference for the non-invasive quantitative assessment of coronary artery function [19].

## 3. Basics of CMR Studies of Myocardial Perfusion

**Fig. 1.**
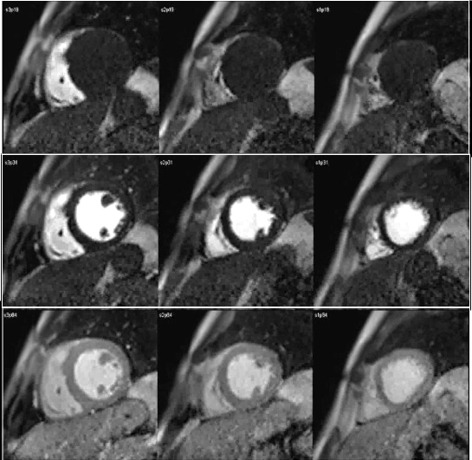
**CMR sequence of myocardial perfusion**. Three simultaneous levels of the left ventricle (LV) are imaged every heart beat for 60 cycles during the first-pass of a contrast agent. Presented here are those frames corresponding to the wash-in of the agent through the right ventricular chamber (top row), then into the left one (middle row), and, finally, through the ventricular myocardium (bottom row), where the perfusion of the heart muscle is visually perceived as a global, homogeneous increase in signal intensity.

The study of myocardial perfusion by CMR was introduced during the early years of the short history of the technique itself [20], and the approach then proposed has not changed essentially. Here, as in nuclear scans, it is the kinetics of a tracer, an MR contrast agent (CA) which is monitored during its first pass through the heart chambers and, finally, the left ventricular myocardium, that enhances its signal as the agent reaches the microcirculatory network of the heart muscle (Fig. 1).

### 3.1 Technical Considerations

CMR sequences to perform such a study must be ECG-gated, fast enough to permit short acquisition time of images, and highly sensitive to the signal intensity (SI) changes of the myocardium induced by the CA. Gadolinium compounds (the universal chemical basis of CAs in MR) diffuse into the extracellular space at their arrival to the myocardial capillary compartment, leading to a reduction of the T1 relaxation time. In consequence, perfusion sequences must be T1 weighted in order to detect the marked increase in SI thus produced. Such a feature is achieved by a saturation-recovery preparation of the sequence with, either, a Gradient-Recalled-Echo (GRE), Steady-State-Free-Precession (SSFP), or Echo-Planar-Imaging (EPI) readout strategy. Of note, 3T systems offer advantages over 1.5T due to their higher signal-to-noise ratio and, when using GRE readouts, also in reducing artifacts [21]. Each one of these approaches, however, implies particular tradeoffs involving relevant imaging parameters, as are SI, spatial coverage and spatial resolution. A detailed discussion on these aspects is not the aim of this review, as has been comprehensively covered elsewhere [22].

**Fig. 2.**
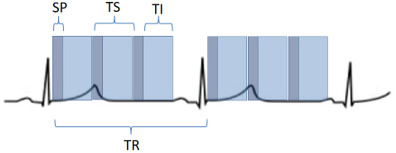
**Scheme of a perfusion sequence with saturation recovery preparation**. SP, saturation preparation; TI, time of imaging readout; TR, temporal resolution; TS, time-per-slice.

There are, however, several concepts upon perfusion sequences which the practitioner must be familiar with. First, on the issue of *temporal resolution*, that is conditioned by the heart rate of the patient, as each slice of the same location is obtained once every heart beat. A different concept is the *time-per-slice*, which is around 130 msec, for conventional Fast-Low-Angle-Shot (FLASH) or SSFP sequences, of which nearly 90 msec are consumed in the actual readout (*time-per-image*) [22]. This, together with the heart rate, limits the number of slices to be acquired every cardiac cycle, or *spatial coverage*, which is usually limited to 3, or 4, when the heart rate is below 80 beats/min, and, as the slices are obtained sequentially, each one corresponds to a different phase of the cardiac cycle (Fig. 2). Finally, the in-plane *spatial resolution* provided by these sequences lies between 2 and 3 ${\mathrm{mm}}^{2}$. Under these conditions, it is assumed that the sequence provides adequate coverage of the left ventricular myocardium and appropriate image resolution for the visualization of the first-pass of a dose of CA given intravenously. The perfusion sequence is started simultaneously with the CA administration and is prescribed to last for 50–60 heart beats in order to cover for both the wash-in and wash-out of the agent through the heart chambers and, particularly, the left ventricular myocardium. The introduction of strategies of automatic motion correction, based on separation of SI changes and motion components of images [23], have made possible the obtention of perfusion sequences during free breathing, which greatly improves both the patient acceptance and the operator reliance on the visual inspection of images.

### 3.2 Rest and Stress Studies

**Fig. 3.**
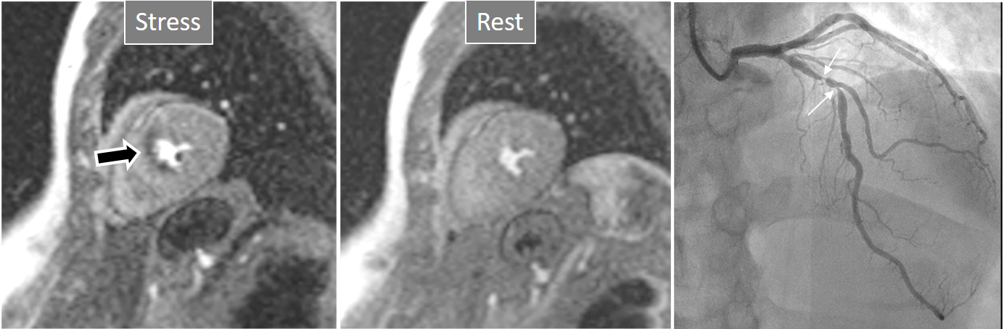
**Induced perfusion defect**. Subendocardial perfusion defect at the interventricular septum during stress (left panel, black arrow), not present at rest (middle panel), in a patient with significant coronary stenoses in the left anterior descending (LAD) artery (right panel, white arrows).

The aim of perfusion studies is the demonstration of a blunted increase—or a reduction—in regional MBF in response to a vasodilator stimulus which can be attributed to a flow-limiting coronary artery lesion. Rest perfusion studies alone are not useful in this sense, as basal MBF is kept within normal limits even in territories supplied by a severely stenosed vessel, and is only after a vasodilator agent has been given that the reduction in CFR can be evidenced [24]. In CMR studies, this situation is detected as a reduced increase in SI of the underperfused territories (Fig. 3).

The preferred stressor agent is adenosine, an endogenous purine nucleoside with multiple fundamental physiological effects [25], as is the autoregulatory process of MBF delivery in case of increased oxygen demands. When used as an intravenous drug, either pure or in the form of adenosine triphosphate, induces an effective coronary vasodilatory action after 2–3 minutes of continuous infusion and, due to its very short half-life, of 20–30 seconds, its action ceases shortly after interrupting the infusion. The systemic and coronary vasodilatory effect is due to its binding to specific receptors (A2a) [26], although adenosine also activates other receptors, as A1, causing negative dromotropic effects at the level of the AV node, or A2b, which induces bronchial constriction. For this reason, the main unwanted side effects of the drug are transient AV blockade and bronchospasm [27], and while the first one is often unpredictable, in the absence of pre-existent AV conduction delay, bronchospasm can be anticipated to occur in patients with antecedent severe asthma or a recent bronchial infective process, who should not receive adenosine. Other minor side-effects, however, are quite frequent (>80%), including flushing, dyspnea (usually without objective deoxygenation), cough, headache, and chest or gastric discomfort, all of them quickly vanishing after the end of the infusion. As a general rule, side-effects are more likely to appear in young, overweighted females [27].

**Fig. 4.**
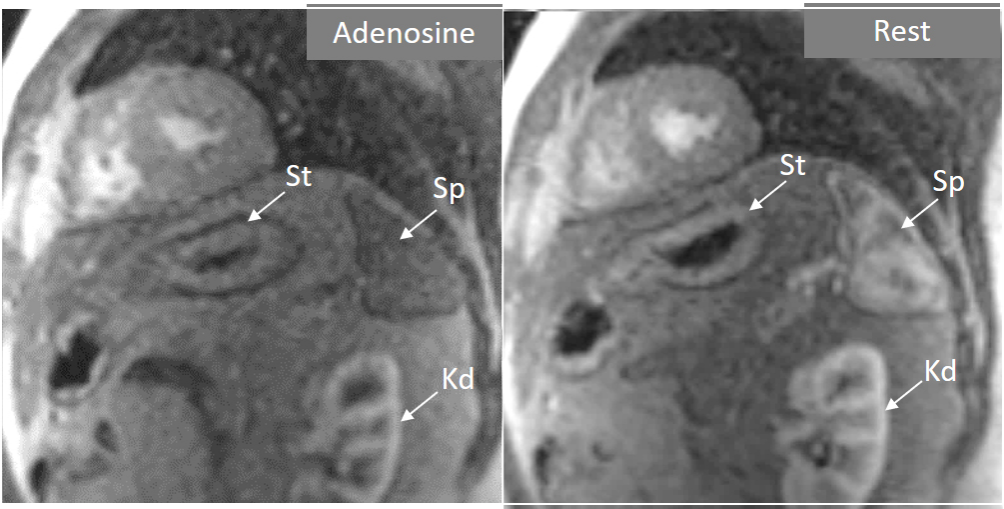
**Splenic switch-off**. Stress (left panel) and rest (right panel) perfusion studies showing dark signal intensity of the spleen (Sp) during adenosine infusion, that turns into an intense bright signal at rest, where trabeculae and splenic pulp compartments are readily visualized. This contrasts with the stomach wall (St) and the left kidney (Kd), that exhibit similar degree of contrast arrival both at stress and at rest.

The presence of these side effects is, in fact, a reliable sign of an adequate vasodilatory response in a particular patient. An appropriate vasodilatory effect of adenosine is not always accomplished with the recommended dose (140 μg/kg/min for 4 minutes), and suboptimal vasodilation is judged to involve up to a 16% of patients [28], with the potential for a false negative result of the perfusion study. Besides clinical symptoms, there are some changes in physiological parameters that can be evaluated as indicators of an adequate response. Thus, it is estimated that an increase in 10 bpm of heart rate and a decrease in 10 mm Hg in systolic blood pressure are reliable signs in this sense. A suboptimal vasodilatory effect of adenosine is hardly predictable and, for this reason, close monitoring of the patient during the infusion of the drug is mandatory, including heart rate, blood pressure, and blood oxygen saturation. As has been reported [29], in the absence of signs of peripheral hemodynamic response, increasing the dose of infusion of the agent to 170 and, if necessary, up to 210 μg/kg/min, is safe and can surmount the inadequate effect of adenosine at lower doses. A useful sign of an appropriate effect of adenosine is the so-called “splenic switch-off” [30] which consists on a blunted increase in signal intensity of the spleen during adenosine infusion, in contrast with the rest study (Fig. 4), and due to a vasoconstrictive effect of the A1 and A2b receptors in the splenic artery. The presence and intensity of the splenic switch-off has been studied at different, increasing dosages of adenosine [31], showing that at 175 μg/kg/min a significantly higher proportion of patients presented with the sign, in comparison with the usual standard dose of 140 μg/kg/min, suggesting a more adequate coronary vasodilatation at this higher dosage of the agent. Obviously, this sign is useful only retrospectively, as the stress perfusion study must have been completed in order to assess the splenic signal intensity.

Other vasodilator agents available, with mechanisms of action related with adenosine, include dipyridamole and regadenoson. While the first acts indirectly, by blocking adenosine reuptake and increasing the levels of endogenous adenosine, regadenoson is a highly selective A2a adenosine receptor agonist. In consequence, dipyridamole has a similar rate of unwanted effects to adenosine while regadenoson has an effect restricted to coronary vasodilation, with negligible broncho-constrictive effect [32]. Of note, regadenoson has no effect upon splenic blood flow and, thus, no changes on splenic perfusion can be expected in comparison with rest studies [30]. An important advantage of regadenoson is that it can be given as a single bolus of a fixed dose (400 μg), with a peak of action within the first minute. This simplifies the logistics of the study: while 2 independent venous lines are required when adenosine or dipyridamole are used (one for the continuous infusion of the drug and the second one for the CA), regadenoson is given as a bolus before the CA injection, and only one line is required. Comparison of these 3 agents [33] has shown similar vasodilatory action for adenosine and regadenoson and a less prominent effect of dipyridamole.

### 3.3 Strategy of CMR Exams for IHD Including Perfusion Studies

The perfusion study is a module within a CMR exam that, besides the evaluation of ventricular volumes and function, covered by cine-MR sequences, and that on myocardial tissue characterization, by means of late gadolinium enhancement (LGE) sequences, provides a comprehensive body of information upon a patient with proven or suspected IHD.

**Fig. 5.**
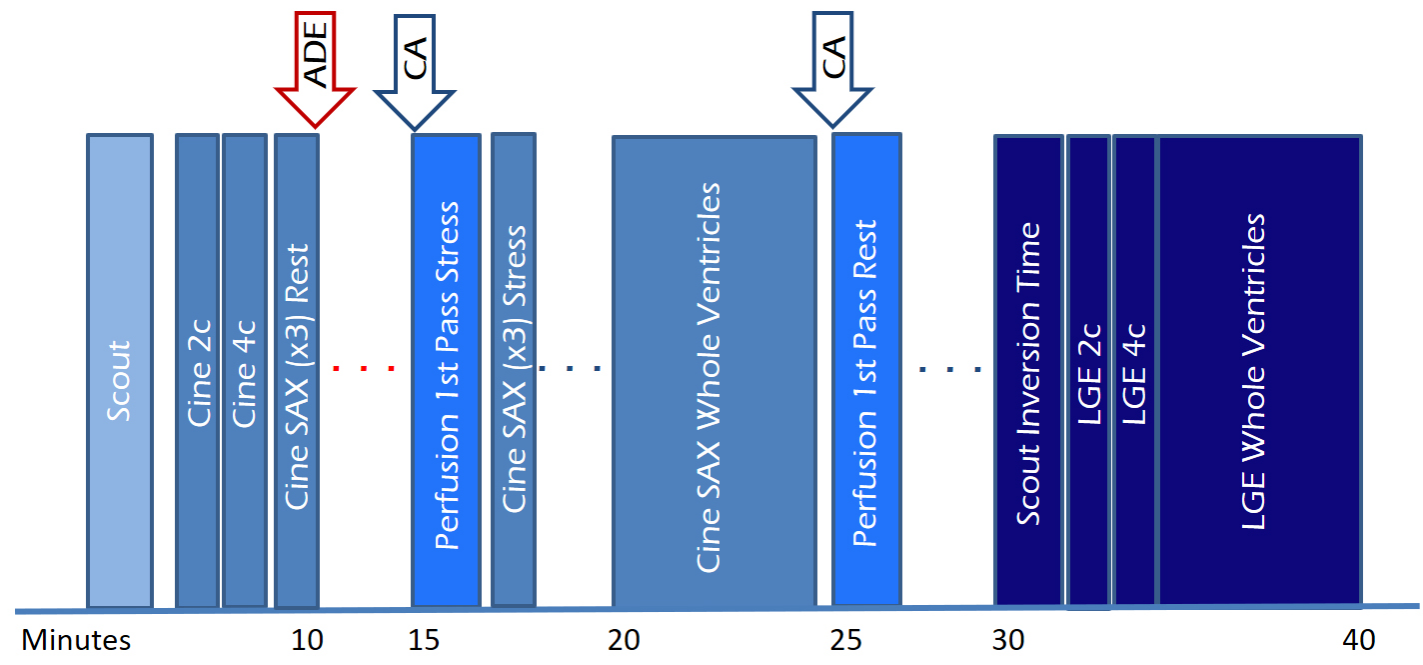
**Study protocol of a comprehensive CMR study of ischemic heart disease, including function, perfusion and tissue characterization modules with an estimated timeline**. 2c, two-chamber; 4c, four-chamber; ADE, adenosine; CA, contrast agent; LGE, late gadolinium enhancement; SAX, short-axis.

An appropriate ordering of these 3 modules results in an efficient study protocol in terms of examination time. Scientific societies have issued detailed recommendations on this issue [34]. One of such optimized protocols is presented in Fig. 5. Of note, this study planning includes a set of 3 cine sequences oriented in short-axis (SAX) which is obtained both at rest and repeated immediately after the stress perfusion, while adenosine is still flowing. The rationale for including rest and stress function studies lies in the proven adverse prognostic effect of an induction of regional contractile function in addition to the perfusion defect, being thus a potential marker of a particularly severe instance of inducible ischemia [35, 36].

The rest study, which is performed 10 minutes apart from the stress, has been deemed as irrelevant by authorized voices [37, 38] on the basis of its scarce contribution to diagnostic accuracy when perfusion sequences are visually analyzed. While this can be true in cases with a definitely normal stress study, the resting one is still on use in many departments where it is considered useful as a comparative reference when there are doubts on a potential defect in the stress study, or when perfusion defects at rest are predictable, as discussed below. Also, when semi-quantitative or fully quantitative perfusion analysis are performed, the rest study is necessary for calculation of CFR values.

## 4. Interpretation of Myocardial Perfusion Studies

**Fig. 6.**
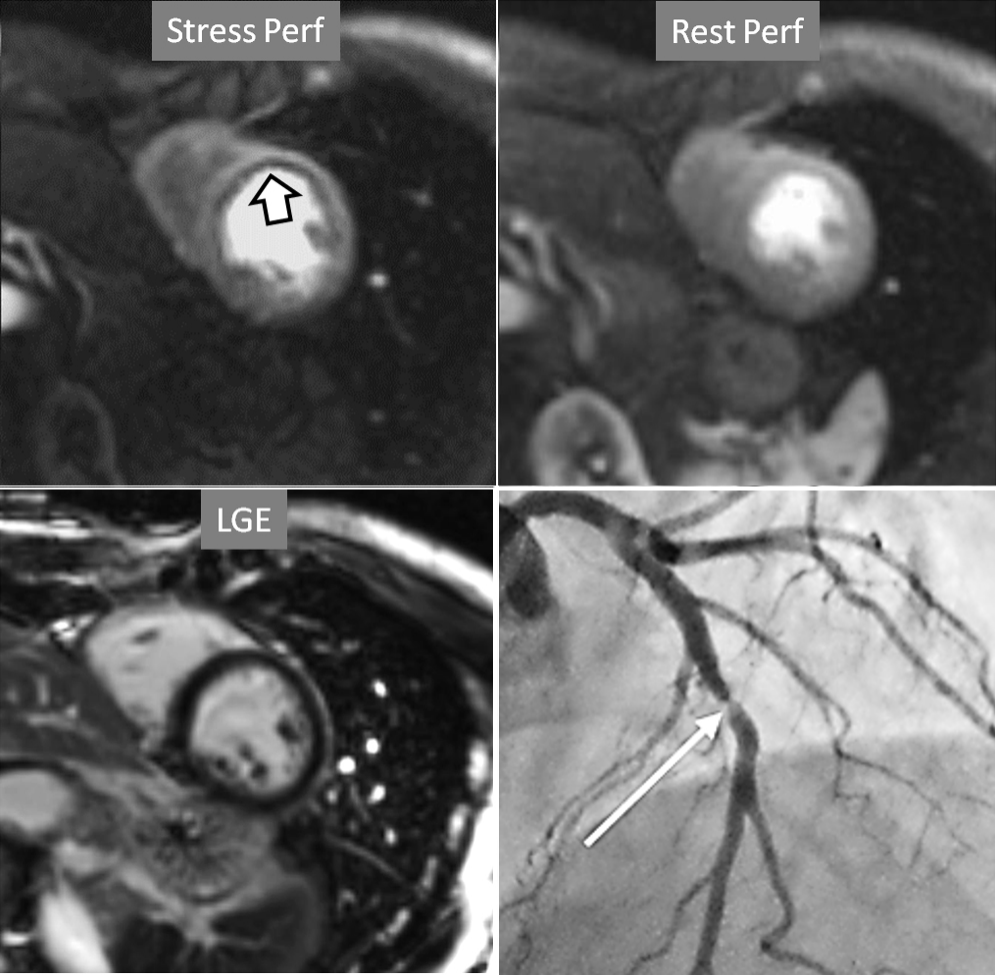
**Single-vessel perfusion defect**. Extensive subendocardial perfusion defect at the antero-septal region (arrow, on the upper left panel) not present at rest, in a region without LGE (lower left panel). Angiography proves this defect to be due to a tight stenosis of the LAD coronary artery (arrow, on the lower right panel).

The most immediate and commonest method of analysis is the visual detection of a distinctive deficient increase in signal intensity of a region of the left ventricular myocardium during the first passage of a CA bolus under vasodilatory conditions lasting for at least 3 consecutive frames of the sequence. This perfusion defect should not be present at rest and should not correspond to an area of previous MI. The detection of such a defect is assumed to be due to a reduction in CFR of this territory which, when attributed to a particular coronary artery [39], allows for the conclusion of the presence of a significant epicardial stenosis in the vessel (Fig. 6).

Criteria for an appropriate interpretation of dark, unenhanced regional myocardial signals as due to perfusion defects have been issued by scientific societies [40]. According to these recommendations, it is accepted that a true defect (1) appears when contrast arrives at the left ventricular myocardium, (2) persists for several cardiac cycles, (3) is not restricted to a thin linear contour, (4) it is more prominent at the subendocardial level, extending variably through the whole thickness of the myocardium, (5) it is not present at rest, and (6) corresponds to a distribution territory of a coronary artery.

**Fig. 7.**
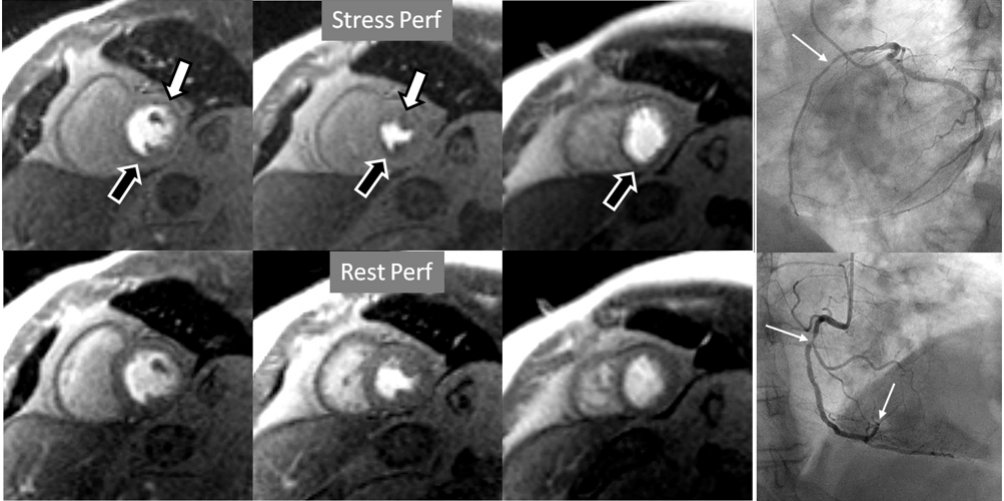
**Two-vessel perfusion defect**. Stress perfusion defects at the anterior (white arrows) and inferior (black arrows) walls, not present at rest. Angiography (right panels) shows significant stenoses (white arrows) of the LAD (upper panel) and the right coronary artery (RCA) (lower panel).

**Fig. 8.**
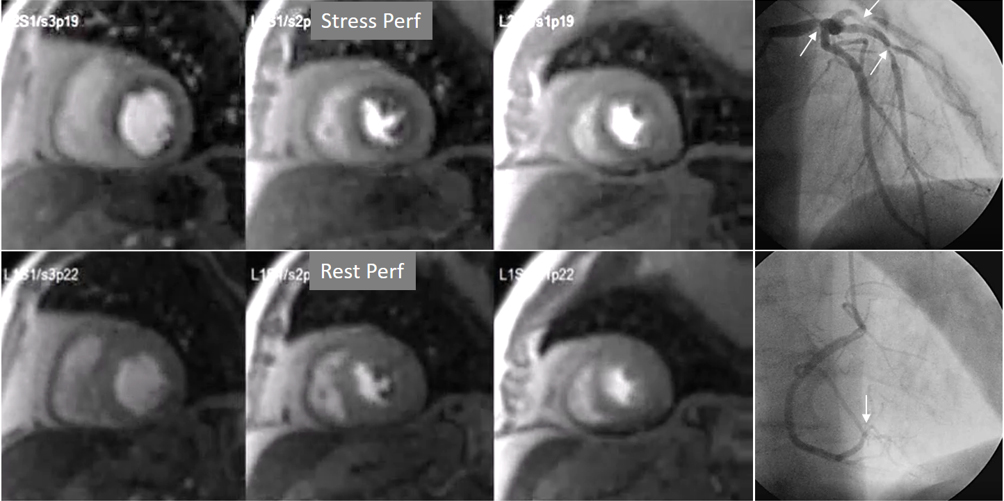
** Three-vessel perfusion defect**. Stress and rest studies in a patient with severe left main and 3-vessel CAD (arrows, on the right panels) showing an inducible perfusion defect in most, if not all, of the myocardial segments during vasodilatory stress.

Perfusion defects in different territories can be detected in case of multivessel disease (Fig. 7) and, in patients with severe 3-vessel CAD, the perfusion study may show a global, intense, persistent defect which is readily recognizable (Fig. 8).

**Fig. 9.**
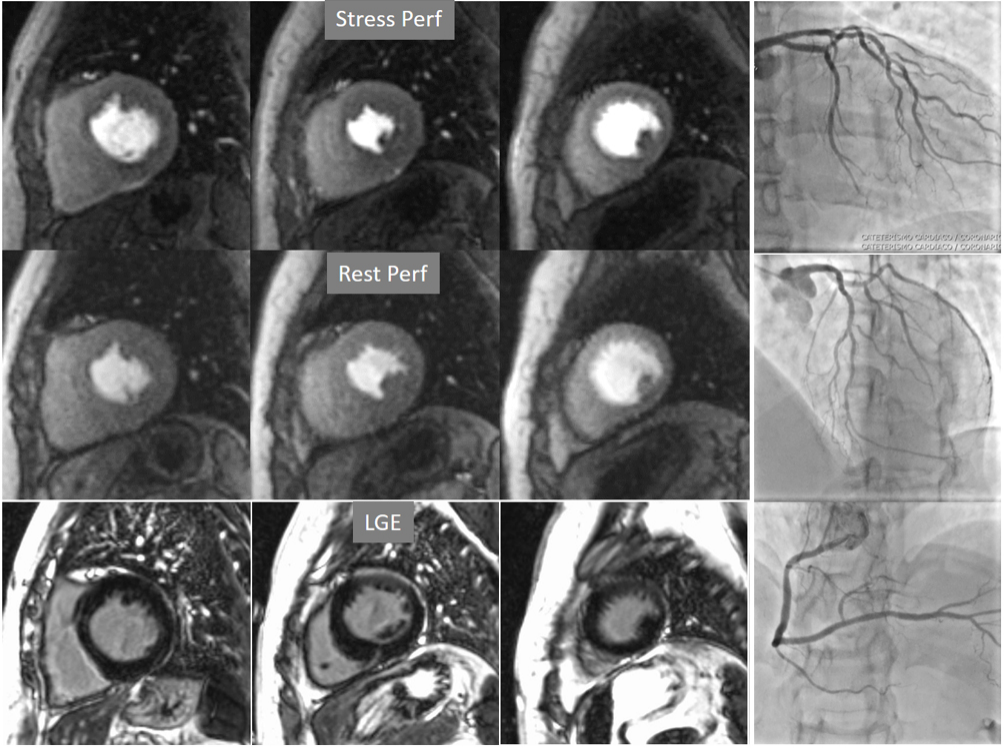
**Microvascular defect**. Circumferential subendocardial perfusion defect at stress, not present at rest, in the absence of LGE, in a patient without significant epicardial coronary lesions at angiography (right panels).

**Fig. 10.**
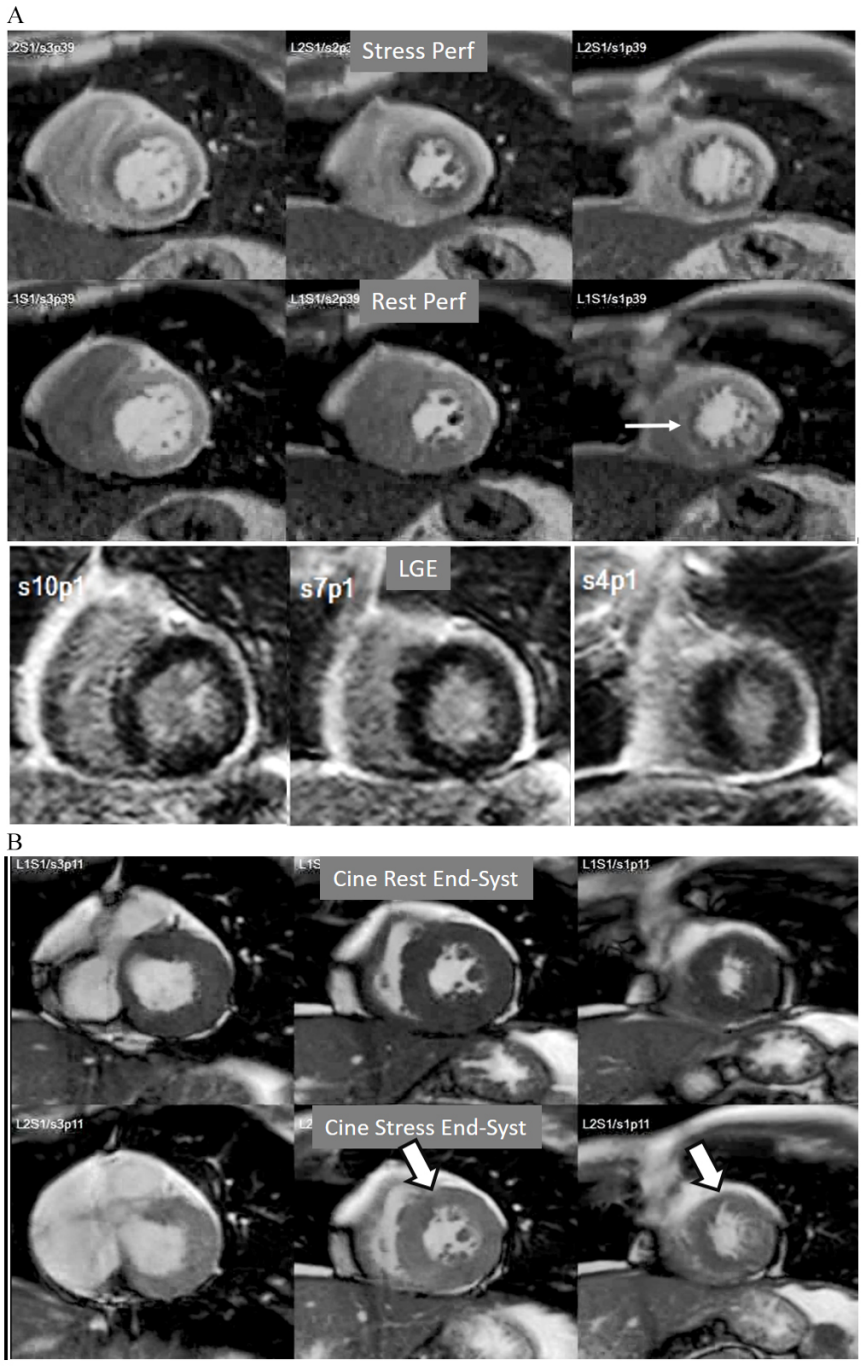
**Multi-vessel perfusion defect**. (A) Global stress hypoperfusion in a patient with multivessel CAD. Observe the non-uniformity of the defect, with different degrees of intensity and transmurality and, also, with the presence of a defect at rest (arrow), in the absence of LGE. (B) End-systolic frames from cine studies at the same level of the perfusion slices showing inducible antero-septal hypokinesia during stress (arrows).

A not infrequent finding is a diffuse, annular, transient, subendocardial defect not present at rest (Fig. 9), which, in the absence of any recognizable obstructive lesion in an epicardial coronary artery, has been deemed to correspond to diffuse microvascular dysfunction (MVD) [41, 42]. CMR perfusion is sensitive to both the downstream effects of discrete epicardial stenoses and those due to disturbances of coronary microcirculation, and subendocardial hypoperfusion is on the pathophysiological basis of each one. For this reason, theoretically, a global, inducible subendocardial defect, could also be due to multivessel epicardial disease with balanced ischemia. In practice, however, this latter instance presents with extensive but heterogenous defects in terms of intensity, persistence and transmurality and, not rarely, also with perfusion defects at rest (Fig. 10A). Importantly, an accompanying induced contractile disfunction may be found in particularly impaired regions (Fig. 10B), a finding which is never seen in MVD.

**Fig. 11.**
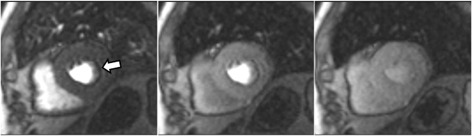
**Perfusion artifact**. Frames from a single slice perfusion study selected at different time points: a dark-rim artifact (arrow, in the left panel) is already seen at the time of contrast arrival to the left ventricular chamber, persists during the actual myocardial perfusion (middle panel), and vanishes shortly afterwards (right panel).

**Fig. 12.**
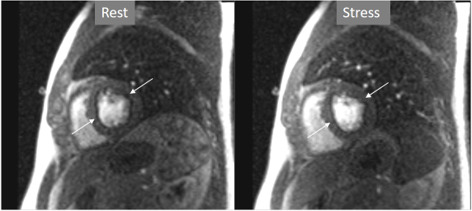
**Perfusion artifact**. Dark-rim artifact present at both stress and rest studies (arrows).

**Fig. 13.**
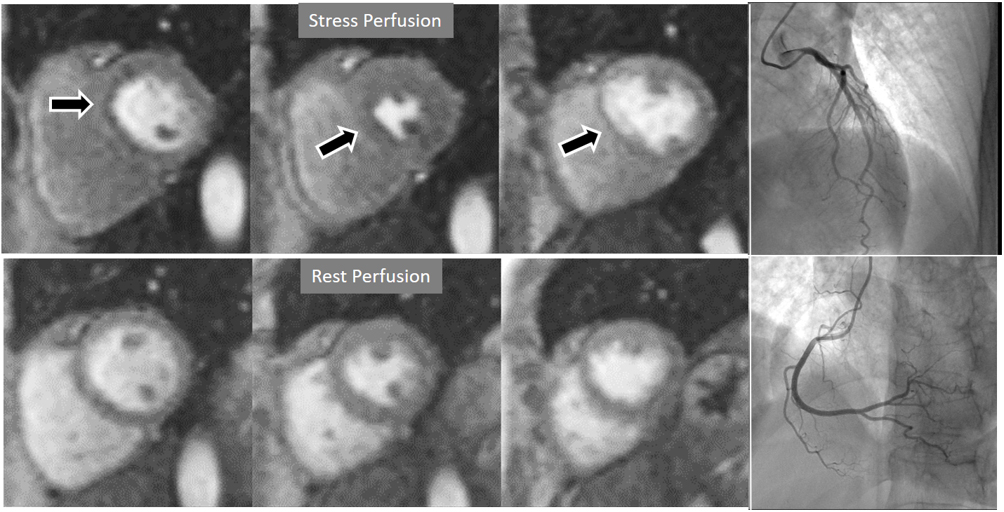
**Perfusion artifact**. Artifactual stress perfusion defect (arrows) not present at rest, leading to angiography which did not show CAD (right panels).

Important for a reliable identification of perfusion defects is the recognition of artifacts that may mimic a true defect which, unfortunately, are frequent, even with advanced MR systems. The most relevant is the so-called “dark-rim artifact”, which consists on a subendocardial, sharp linear signal of very low intensity that is due to magnetic susceptibility effect, to spatial resolution, and/or to motion effects [43, 44]. This dark band may appear as soon as the contrast arrives to the left ventricular chamber, is usually very thin (1–2 pixel wide), transient, and can be present at both the rest and stress studies [40] (Figs. 11,12). Despite these hints, cases where an artifact occasionally challenges the judgement of the observer still persist (Fig. 13).

**Fig. 14.**
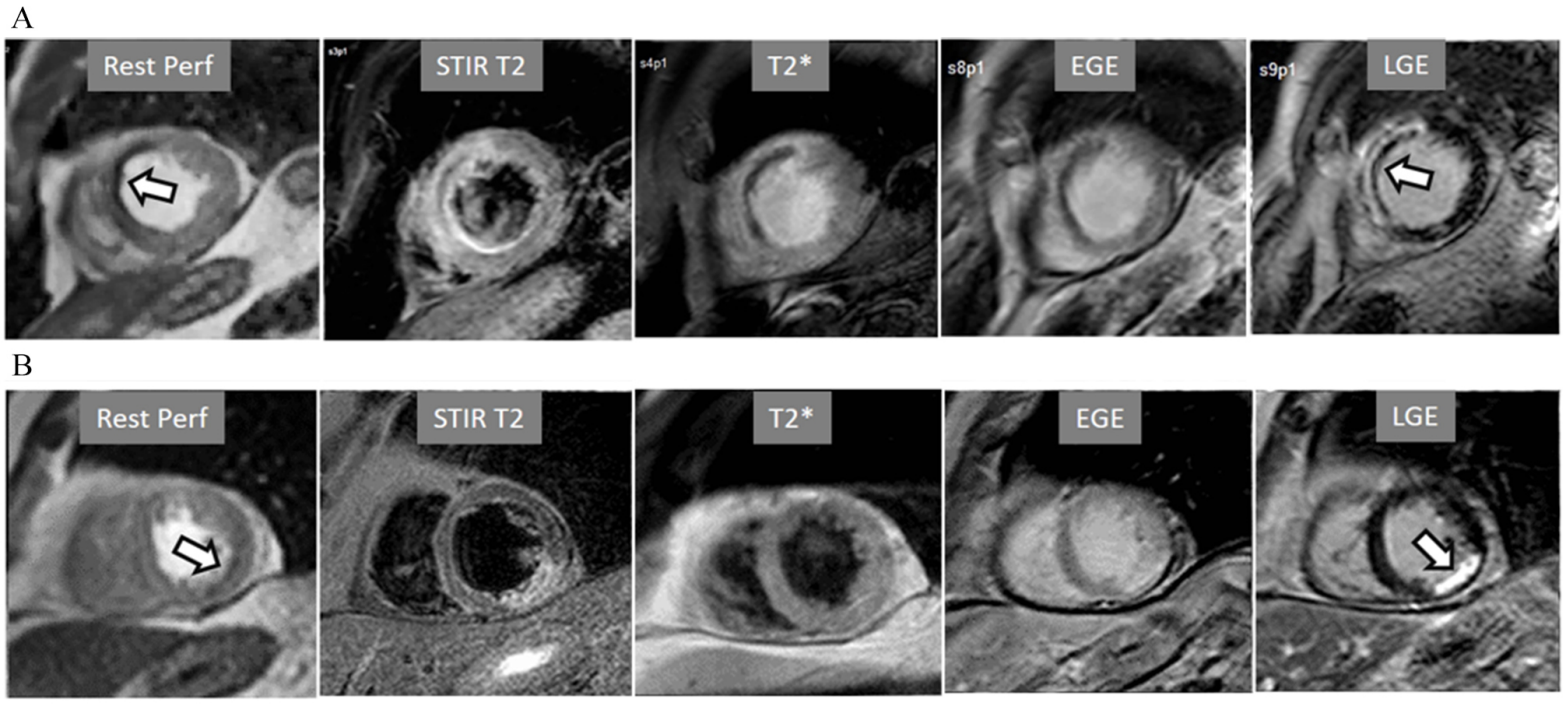
**Rest perfusion after acute myocardial infarction**. (A) Frames from different sequences in a case of acute MI showing, from left to right, (1) gross transmural defect at the rest perfusion study (arrow); (2) signs of regional myocardial edema at STIR T2, with a mid-line low intensity region which proves to be due to (3) intramural hematoma at T2* sequence; (4) persistence of hypoperfusion at Early Gadolinium Enhancement (EGE); and (5) transmural anteroseptal necrosis with a subendocardial area of microvascular obstruction (arrow). (B) The same series of sequences in another patient with AMI showing (1) mild subendocardial defect at rest (arrow); (2) regional edema; (3) absence of hematoma at T2*; (4) lack of persistent defect at EGE; and, finally, (5) a transmural infero-lateral LGE (arrow) without microvascular obstruction.

**Fig. 15.**
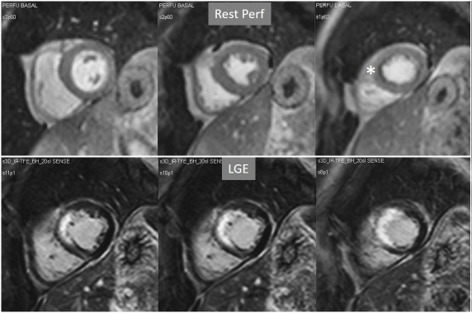
**Rest perfusion in chronic myocardial infarction**. Basal rest perfusion study showing a reduced subendocardial septal defect (asterisk) at the apical septum in a patient with an extensive old anteroseptal MI evidenced at LGE.

Particularly important, in practice, is the issue of perfusion studies in patients with previous MI. In theory, a basal rest perfusion study should exhibit a reduced signal intensity in the infarcted area, as is the case in the acute phase of a large transmural MI (Fig. 14A). However, not infrequently, the presence and extent of a rest perfusion defect does no correlate with the actual area of necrosis in either, acute (Fig. 14B) or chronic MI (Fig. 15). Signal intensity in areas of previous MI is thus, not dependent merely on the presence of scar tissue, but some other factors lead to differences in CA concentration between MI and remote regions and, in this sense, rest perfusion CMR cannot be equated to SPECT studies, where rest defects are directly related to the presence of infarcted myocardial tissue not amenable to radiotracer uptake [45].

**Fig. 16.**
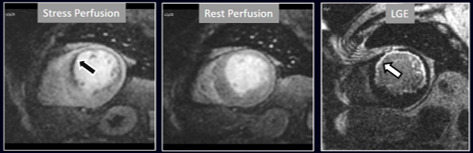
**Stress/Rest perfusion in chronic myocardial infarction**. Perfusion defect during stress (performed in first place) (black arrow), not present at rest, matching the area of LGE (white arrow) in a case of large anterior subendocardial MI.

**Fig. 17.**
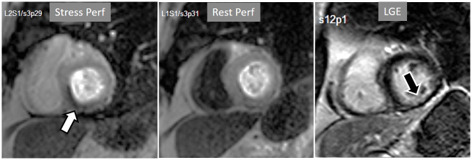
**Stress/Rest perfusion in chronic myocardial infarction**. Perfusion defect during stress (performed in first place) (white arrow), not present at rest, fairly larger than the region of LGE (black arrow) in a patient with a subendocardial inferior MI.

The presence of inducible residual peri-infarction ischemia in a territory with a previous MI has been addressed by the consideration of the extension of a stress perfusion defect in comparison with that of the LGE. Those defects with equal size on both sequences would be labelled as “fixed” (Fig. 16), while those perfusion defects exceeding the area of MI would be considered as “partially reversible” (Fig. 17), independently of rest perfusion studies. The diagnostic performance of the latter finding for the diagnosis of a residual significant stenosis in the infarct-related artery has been found to be adequate, though a good deal of cases with apparently fixed defects had also significantly obstructed arteries [46].

**Fig. 18.**
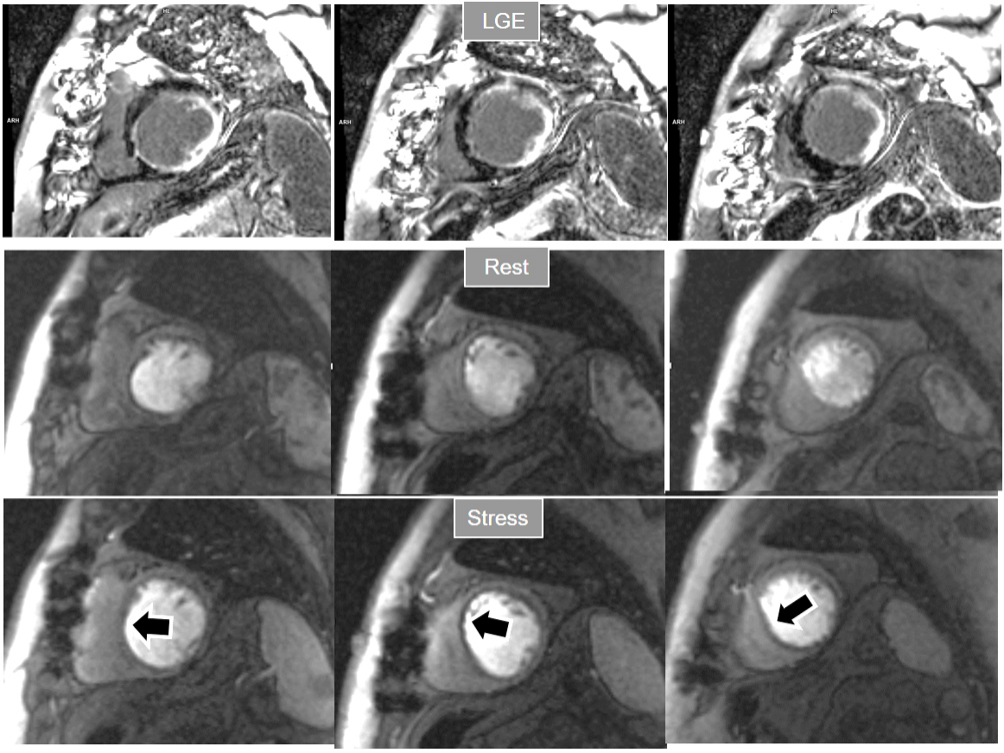
**Rest/Stress perfusion in chronic myocardial infarction**. Patient with previous myocardial necrosis involving most of the LV segments, mainly subendocardial in the anterior and septal regions, and transmural in the lateral wall, as shown in the LGE images (upper panel). The rest perfusion study, performed in first place (middle panel), shows a diffuse subendocardial defect, while, at a subsequent stress study (lower panel), a fair increase in transmurality and extension of the antero-septal defect is seen (arrows) indicating peri-infarction ischemia, while the lateral one remains unchanged. Note the lack of interference of the previous dose of CA on the tissue contrast of the stress study.

**Fig. 19.**
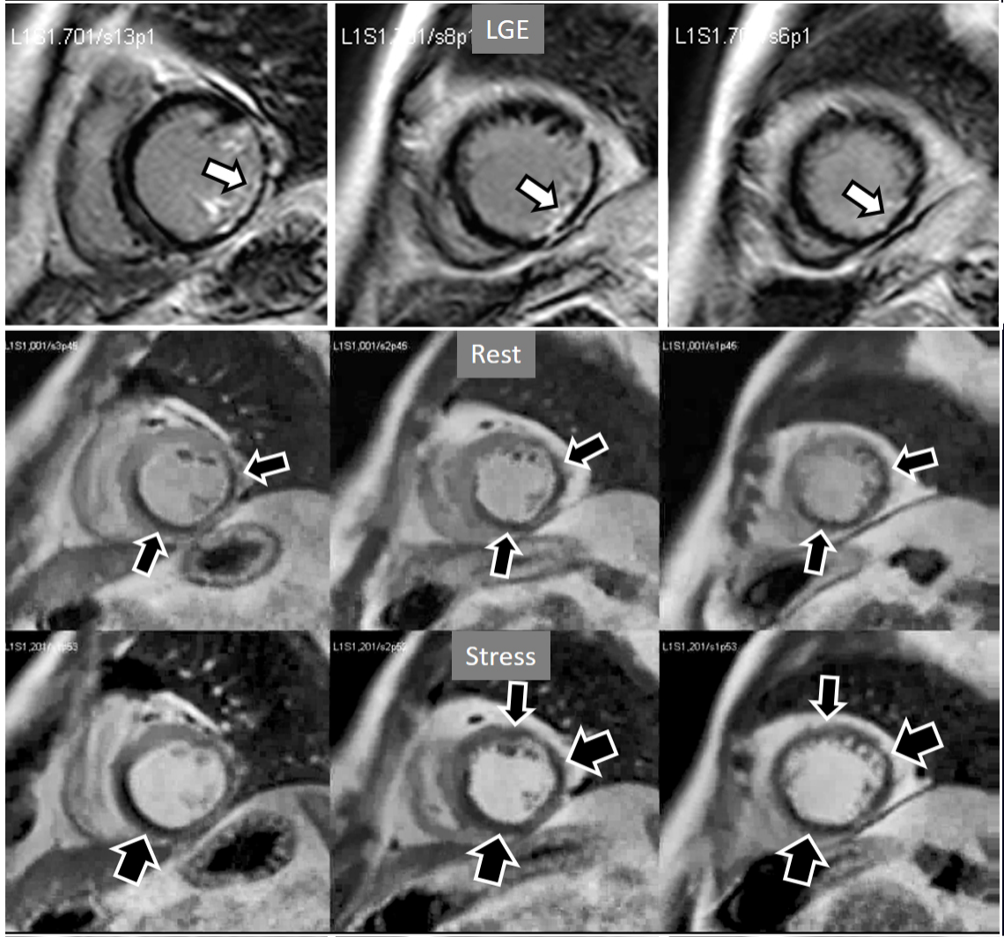
**Rest/Stress perfusion in chronic myocardial infarction**. Upper panel: LGE study showing subendocardial necrosis of the infero-lateral segments (white arrows). Middle panel: rest perfusion study performed in first place showing a defect extending far beyond the limits of the actual MI (black arrows), indicating impaired perfusion at rest of non-infarcted territories (i.e., myocardial hibernation). Lower panel: subsequent stress study showing an increase in extension and transmurality of the inferior defect (large black arrows) and, also, the appearance of and additional induced defect at the anterior wall (small black arrows). Again, the stress study is not interfered by the previous CA administration.

**Fig. 20.**
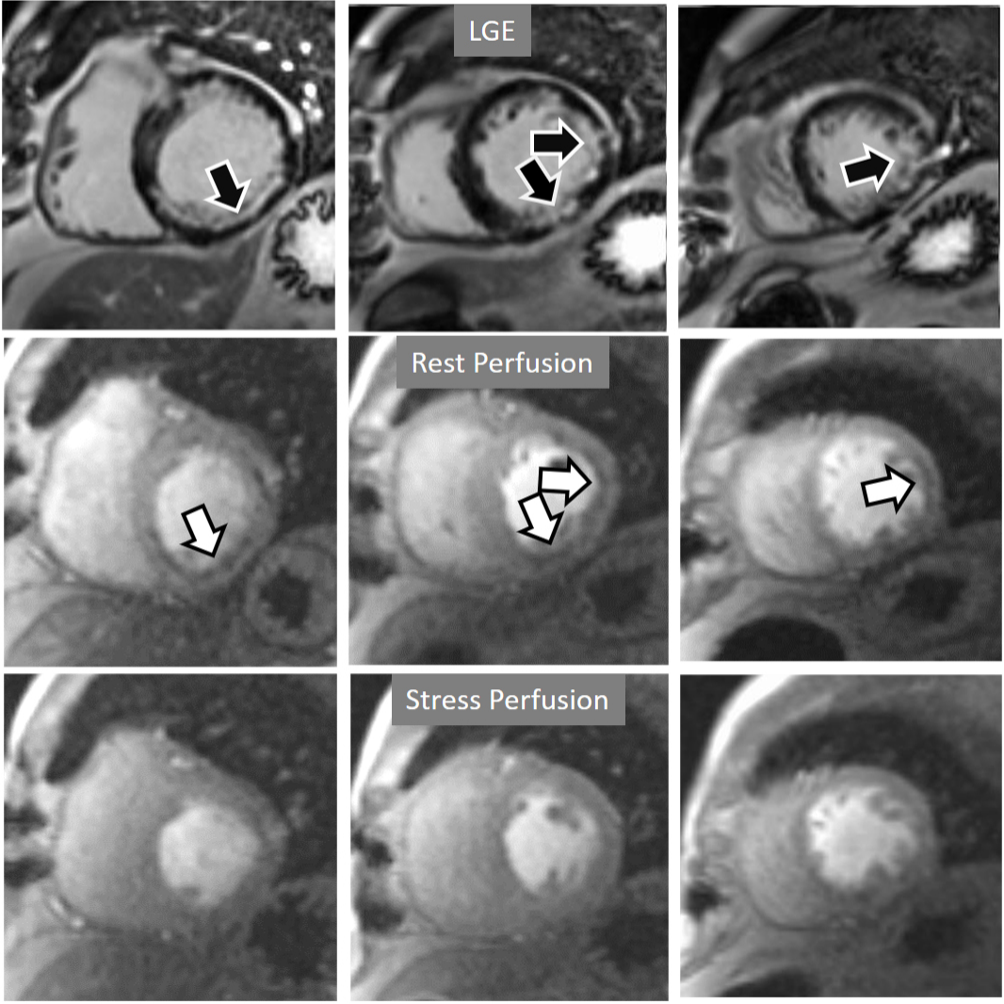
**Rest/Stress perfusion in chronic myocardial infarction**. Subendocardial inferior and infero-lateral MI (black arrows, in the upper panel), leading to a comparable fixed defect at a basal rest study (white arrows, middle panel), and with no apparent defect under stress (lower panel). This latter finding is admittedly due to contamination of residual CA from the previous dose at the infarcted regions. This does not prevent, however, to rule out any newly induced defect.

When considering both, the variability of rest perfusion in the presence of previous MI, and the rather low sensitivity of partially reversible defects, the interest in knowing the actual extension of fixed defects emerges. An option to address the issue is to invert the order of perfusion studies in patients with previous MI, performing the rest study first. This allows for a reliable delineation of the fixed defect that can be easily subtracted from the inducible one, when present (Fig. 18). Moreover, in the case of patients with advanced, severe CAD, in whom a combination of infarcted regions and underperfused territories can be expected, a basal rest perfusion study followed by the stress one is even more helpful (Fig. 19). Certainly, the presence of CA in an area of MI after the first rest perfusion study may contaminate the SI of the second one, but this does not preclude the detection, or exclusion, of newly induced defects (Fig. 20).

## 5. Grading of Inducible Perfusion Defects 

### 5.1 Visual Analysis

The most accessible features in visual analysis of perfusion defects are their extension and degree of transmurality. In consequence, these parameters have been considered in qualitative grading studies including CAD events at follow-up leading to the practical conclusion that moderate or severe ischemia does exist when ≥2 of the 16 myocardial segments, or, more precisely, ≥4 of 32 (considering endo- and epicardial subsegmental regions) present with inducible perfusion defects [47].

**Fig. 21.**
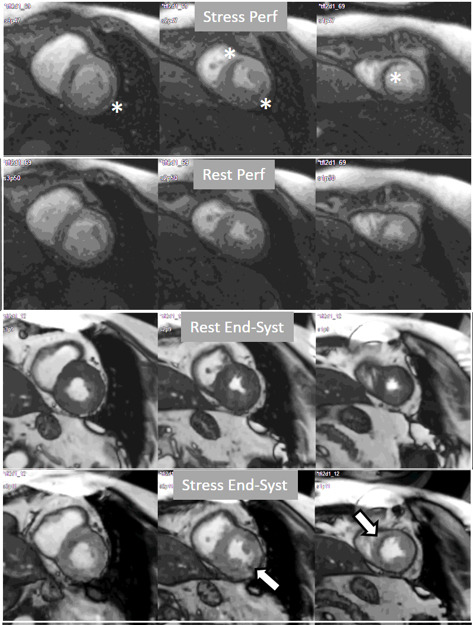
**Grading perfusion defects**. The upper 2 panels present with the very last frames of perfusion studies where a persistent defect is seen at stress in the infero-lateral and antero-septal regions (asterisks). The lower 2 panels show corresponding end-systolic frames with conspicuous contractile adenosine-induced defects (arrows) in both regions.

A study exploring further components of perfusion defects [36] showed that, besides extension and transmurality, also the presence of inducible contractile defect and, particularly, persistence of the defect throughout the full duration of the sequence, were also related with an adverse prognosis (Fig. 21). High values of an ischemic burden score based on these parameters showed prognostic value for clinical improvement after PCI in patients with chronic total coronary occlusion [48].

### 5.2 Semi-Quantitative Perfusion Analysis

**Fig. 22.**
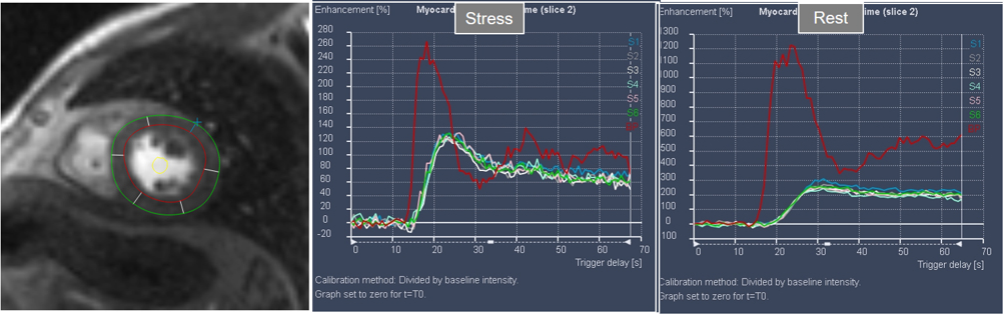
**Semi-quantitative analysis of perfusion**. Contoured segments of the LV from a normal perfusion sequence (left panel) and corresponding SI versus time curves from the blood pool (red line) and the individual myocardial segments during stress and at rest. Observe the tight grouping of myocardial curves and their higher amplitude, earlier time-to-peak and steeper slopes during stress in comparison with those at rest.

**Fig. 23.**
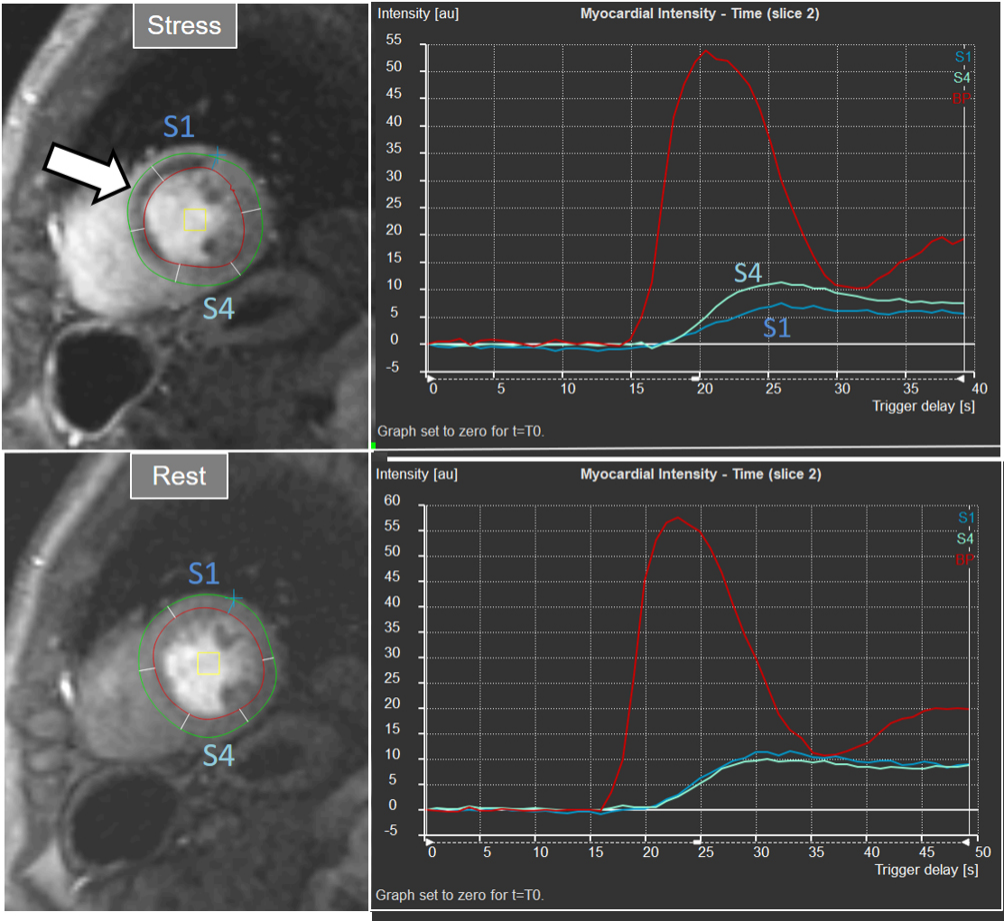
**Semi-quantitative analysis of perfusion**. Inducible defect is visualized in the antero-septal region (arrow). Corresponding curves of SI versus time from blood pool (red) and segments S1 (ischemic) and S4 (normal) (the remaining segments omitted for clarity) show nearly identical upslopes for both segments at rest (lower right panel) while, at stress, a steeper curve for S4 and an actual decrease for S1 are observed (upper right panel). Indexes of myocardial perfusion reserve (normalized for AIF) were 1.5 and 0.65, respectively.

Interest in an objective analysis of myocardial perfusion studies arose early on, investigators focusing on parameters derived from curves of changing myocardial SI over time in perfusion sequences [49, 50] (Fig. 22). The information obtained from SI curves allows for a semi-quantitative estimation of myocardial perfusion as no absolute values of flow are calculated. Parameters such as maximal amplitude, time-to-peak and upslope of the ascending phase of contrast enhancement can be estimated and, from the comparison between vasodilatory stress and rest studies, a relative index of myocardial perfusion is obtained that can be considered as a surrogate of CFR. As myocardial perfusion is dependent on the underlying hemodynamic conditions, a normalization of these values is required for this comparison to be reliable. For this purpose, the time course of the CA in the blood pool can be used as a reference of the so-called “arterial input function” (AIF), to which myocardial parameters are related [51]. Using rest and stress normalized upslope values, a relative MPR index of 1.5 was found to be useful to distinguish between ischemic and normal myocardial segments [52] (Fig. 23).

Despite the proven relationship with impaired coronary blood flow, semi-quantitative estimation of reduced myocardial perfusion faces with important drawbacks in clinical practice, particularly the time required to perform the analysis, which is only partially aided by automatic contour detection methods. Moreover, as with any new measurement, with particular cut-off points, the MPR index thus obtained is not easily adopted by clinical practitioners. For these reasons, methods of semi-quantitative estimation of CMR myocardial perfusion have remained largely into the realm of research.

### 5.3 Quantitative Perfusion Analysis

The aim of quantitative perfusion (QP) is to derive absolute values of MBF from myocardial SI changes through the time lapse of first pass of the CA. Started shortly after the introduction of CMR, several methods were proposed [53, 54, 55] implying cleverly designed strategies, and complex mathematical models, in an effort to circumvent the challenges posed by the technique to a quantitation of MBF, as the dependence of SI changes on the hemodynamic conditions, the assessment of the amount of CA present at the myocardial level, and the non-linearity between the SI and the concentration of the agent.

**Fig. 24.**
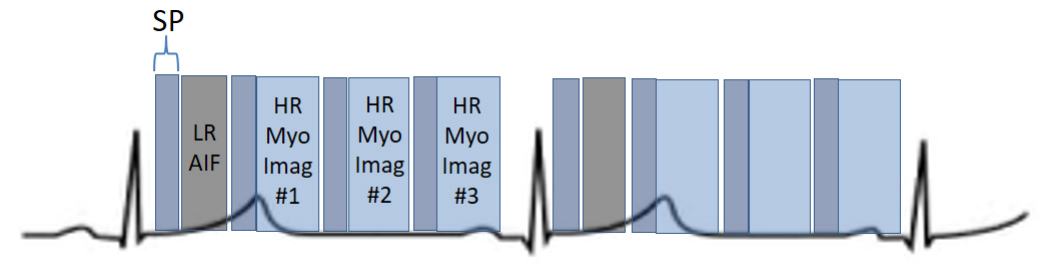
**Scheme of a dual-sequence for QP**. A low-resolution (LR) AIF sequence precedes the set of slices of a high-resolution (HR) sequence for myocardial perfusion imaging. Each image has a saturation recovery preparation (SP).

**Fig. 25.**
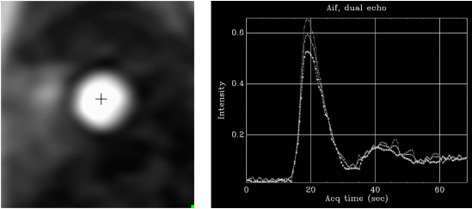
** AIF sequence**. Output single-frame image of the low-resolution sequence with a cross-mark signal indicating where the automatic detection of the LV blood pool is performed (left panel). The resulting plot of raw AIF signals is displayed at right.

**Fig. 26.**
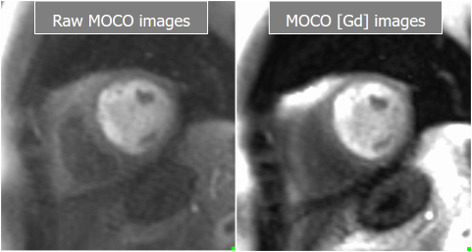
**Basal and Gadolinium-converted perfusion sequences**. Output series from the high-resolution, motion-corrected perfusion sequence before (left) and after (right) conversion to [Gd] units. Both are available for visual analysis.

**Fig. 27.**
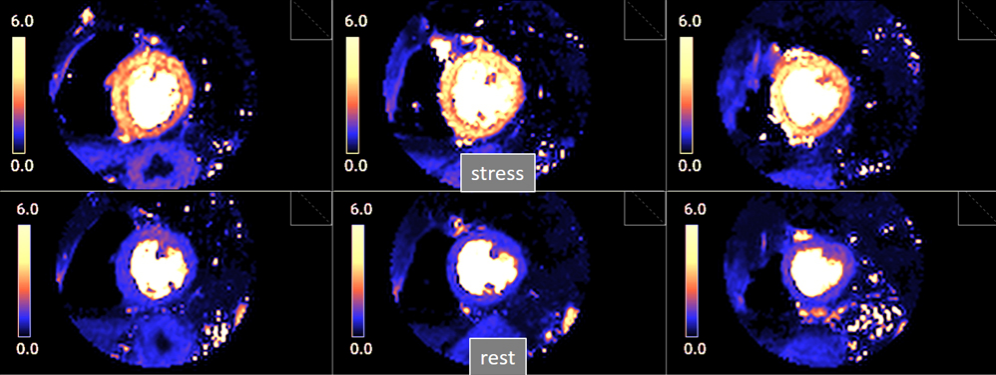
**Pixel-wise perfusion maps**. Maps of MBF (mL/min/g) during adenosine stress (top) and at rest (bottom) in a normal individual. Observe the striking difference between the color parametric maps due to increased values of MBF at stress.

**Fig. 28.**
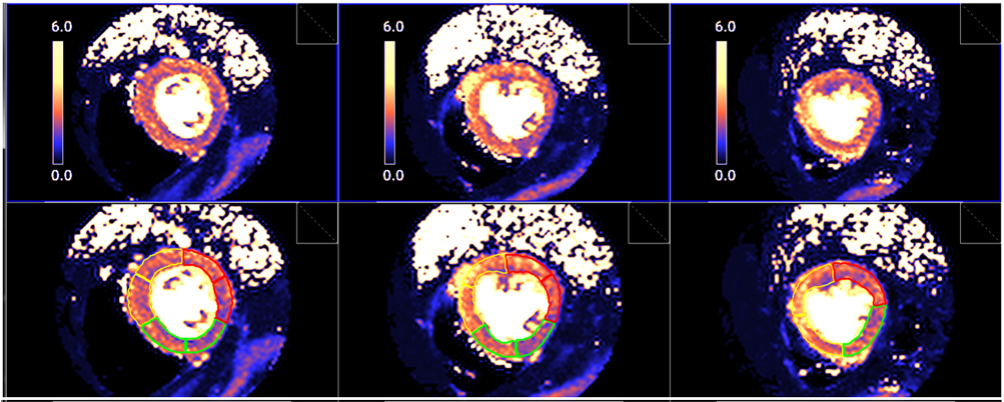
** Segmented pixel-wise perfusion maps**. Stress perfusion map (upper row) and its automatic LV segmentation (lower row).

**Fig. 29.**
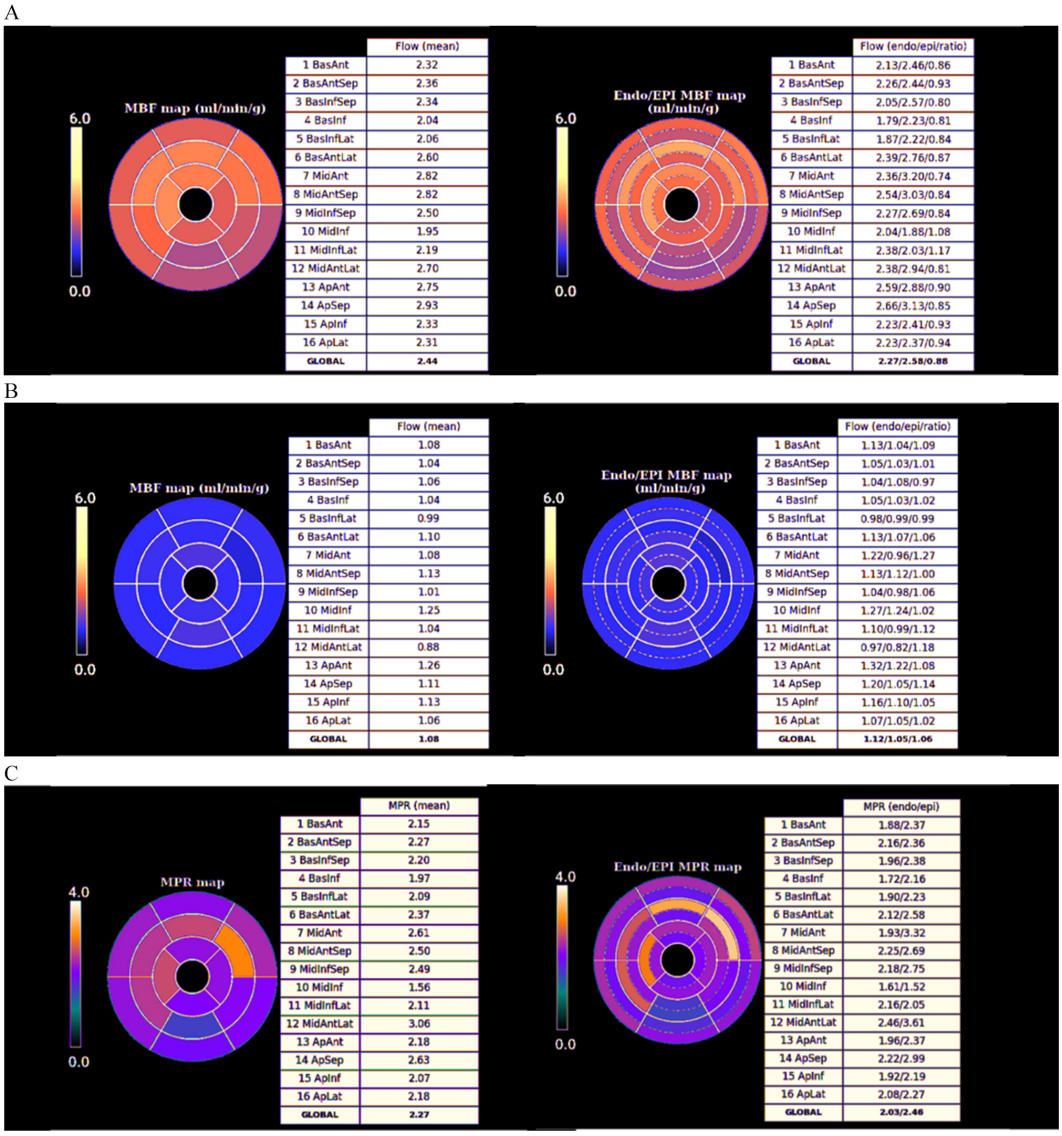
** Bull’s-eye plot segmented values of MBF**. Shown are both, global (left panels) and distributed for endo- and epicardial regions (right panels) at stress (A) and at rest (B). Also, data on the resultant MPR values are presented with the same format (C).

**Fig. 30.**
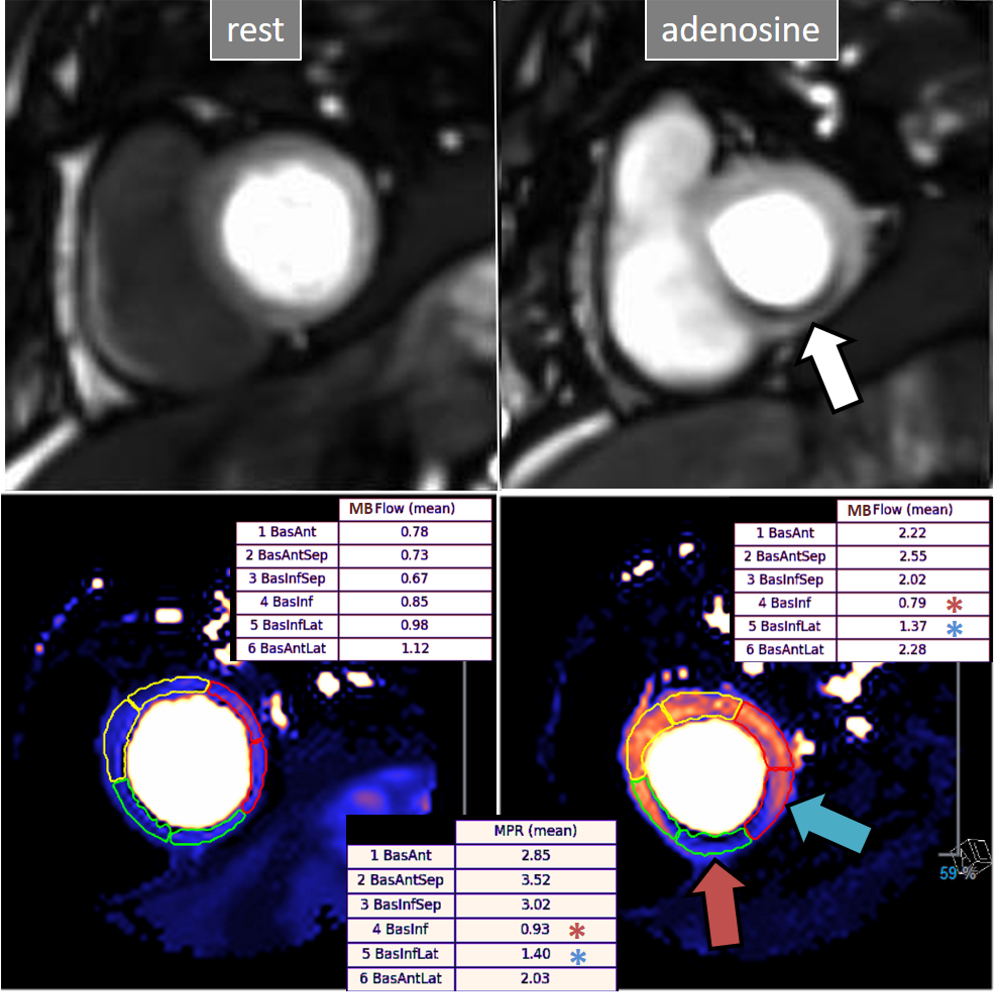
**QP study in a patient with an inducible defect**. A defect at the inferior basal region is detected in the conventional stress perfusion sequence (white arrow). QP maps from the corresponding basal segments show transmural intense reduction of the stress MBF and MPR at the inferior segment (red arrow and asterisks), with less severe involvement of the infero-lateral one (blue arrow and asterisks). Observe the reduced stress MBF in comparison with the rest value of the inferior segment, with a resultant MPR value significantly decreased, inverted actually (0.93), indicating coronary steal phenomenon and, in accordance, probably severe inducible ischemia of the region.

More recently, a new method has been proposed [56] that includes several important solutions contributing to a reliable implementation on the routine workflow of a CMR exam [57]. Firstly, it is based on a dual imaging sequence strategy (Fig. 24), where low-resolution blood pool images used for estimation of the AIF are followed by multi-slice 2D high-resolution myocardial perfusion sequence. The total duration of the scheme is around 500 msec, that allows for the obtention of 3 slices sampled every heart cycle, as long as the heart rate is below 120 b.p.m. AIF extraction from the low-resolution sequence has proven to be reliably obtained automatically [58] (Fig. 25). Second, motion and surface coil intensity correction are applied to both AIF and perfusion images [57, 59]. Third, a process of SI conversion into gadolinium concentration [Gd] units is performed for both sequences [56] (Fig. 26). Fourth, and most important, AIF [Gd] curve and perfusion [Gd] images are inputted into flow mapping step for pixel-wise myocardial flow mapping [57] rendering quantitative values of MBF (in mL/min/g) (Fig. 27). Based on a process of deep learning [60], myocardial segmentation and allocation of flow values are automatically processed (Fig. 28) and integrated into the MRI scanner using the Gadgetron streaming reconstruction software [61, 62]. The final output of the process consists on a bull’s-eye plot of color-encoded MBF values on the LV 16-segment model, and the listed numerical values of absolute flow at stress and at rest and, also, the derived MPR, each of them calculated for the endo- and epicardial halves of the segment (Fig. 29). The presence of inducible perfusion defects is thus detected not only visually on the perfusion color map, but also quantitatively estimated by the absolute values of stress MBF and the corresponding MPR of the involved myocardial segments (Fig. 30). Of note, in case of a suboptimal effect of the vasodilator agent, a lack of increase in stress MFB and MPR values is observed, leading to a potentially false positive diagnosis, in contrast with the visual assessment of perfusion, which, in this case, as stated above, would not show induced defects, with the potential for a false negative.

Importantly, a most remarkable feature of the dual sequence QP is the fully automated image processing and reconstruction which is performed in-line without any user interaction and displayed on the scanner within minutes.

## 6. Validation, Comparison, and Prognostic Studies of Perfusion CMR

**Fig. 31.**
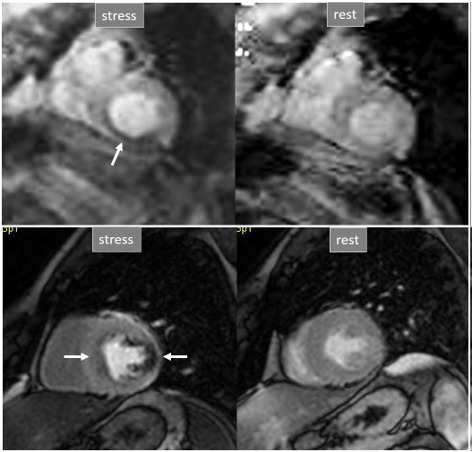
**Improving techniques of perfusion sequences**. Stress and rest studies with inducible perfusion defects (arrows) in exams performed in the early 2000s (upper row) and currently (lower row). Observe the higher image resolution and contrast-to-noise ratio and the absence of movement artifacts in the current sequence.

Studies of validation of visually estimated myocardial perfusion CMR for the detection of significant angiographic coronary stenoses started in the early 2000s with encouraging results [63, 64], despite the limited strengths of the images in comparison with current sequences (Fig. 31). Interest in the technique prompted a good deal of studies with a more reliable standard of reference as is invasive FFR, which have been repeatedly submitted to metanalysis [65, 66], showing pooled values of sensitivity and specificity on the range of 0.90. Qualitative perfusion CMR has shown to compare favorably with SPECT for the diagnosis of CAD in the multicenter study MR-IMPACT [67], a finding confirmed in the landmark CE-MARC study [68], from which a strong predictive value of perfusion for events was also shown [69]. The fairly higher spatial resolution of CMR compared with SPECT (2–3 mm vs 10 mm, respectively) may account for these results [68]. The usefulness of Perfusion CMR for risk stratification and prognosis has been also proven in other multicentric studies [70, 71, 72], and even shown to be noninferior to invasive FFR in decision-making upon revascularization in patients with CAD randomly assigned to either CMR or FFR diagnostic strategies [73]. A derived benefit is the favorable cost-effectiveness profile of those protocols where CMR is introduced in the diagnostic workup [74], a finding supported by data from large, “real world” registry data showing advantages in this respect for CMR in comparison with other techniques [75].

Semi-quantitative methods of analysis of CMR perfusion have also a well-founded basis on early studies of comparison with PET [76], and have shown similar results to qualitative methods in meta-analyses of pooled data [77]. Also, a proven prognostic value has been found in follow-up studies [78] but, as stated above, semi-quantitative analysis techniques have not entered the field of clinical practice due to the complexity of measurements they require.

**Fig. 32.**
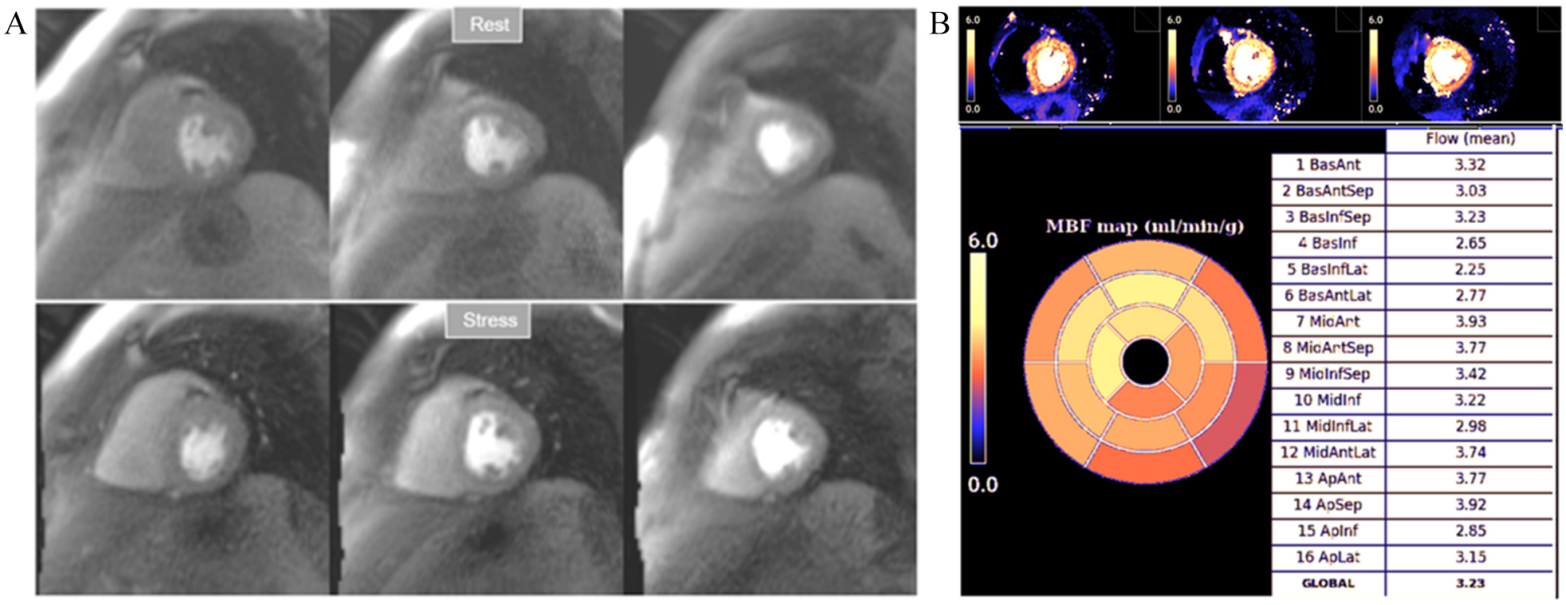
**Normal perfusion mapping**. (A) Rest and stress perfusion studies showing absence of induced visual defect. (B) Normal global and segmental stress MBF.

**Fig. 33.**
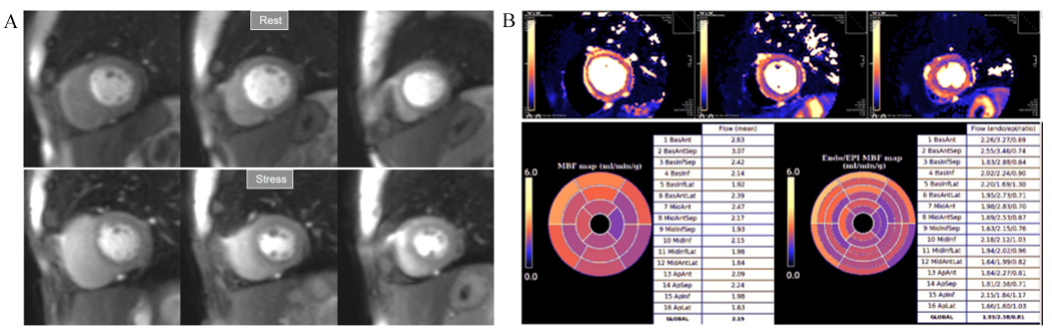
**Mildly abnormal perfusion mapping**. (A) Rest and stress perfusion studies showing absence of induced visual defect. (B) Mildly reduced global stress MBF (left table), particularly in the subendocardial regions (right table), suggesting MVD.

**Fig. 34.**
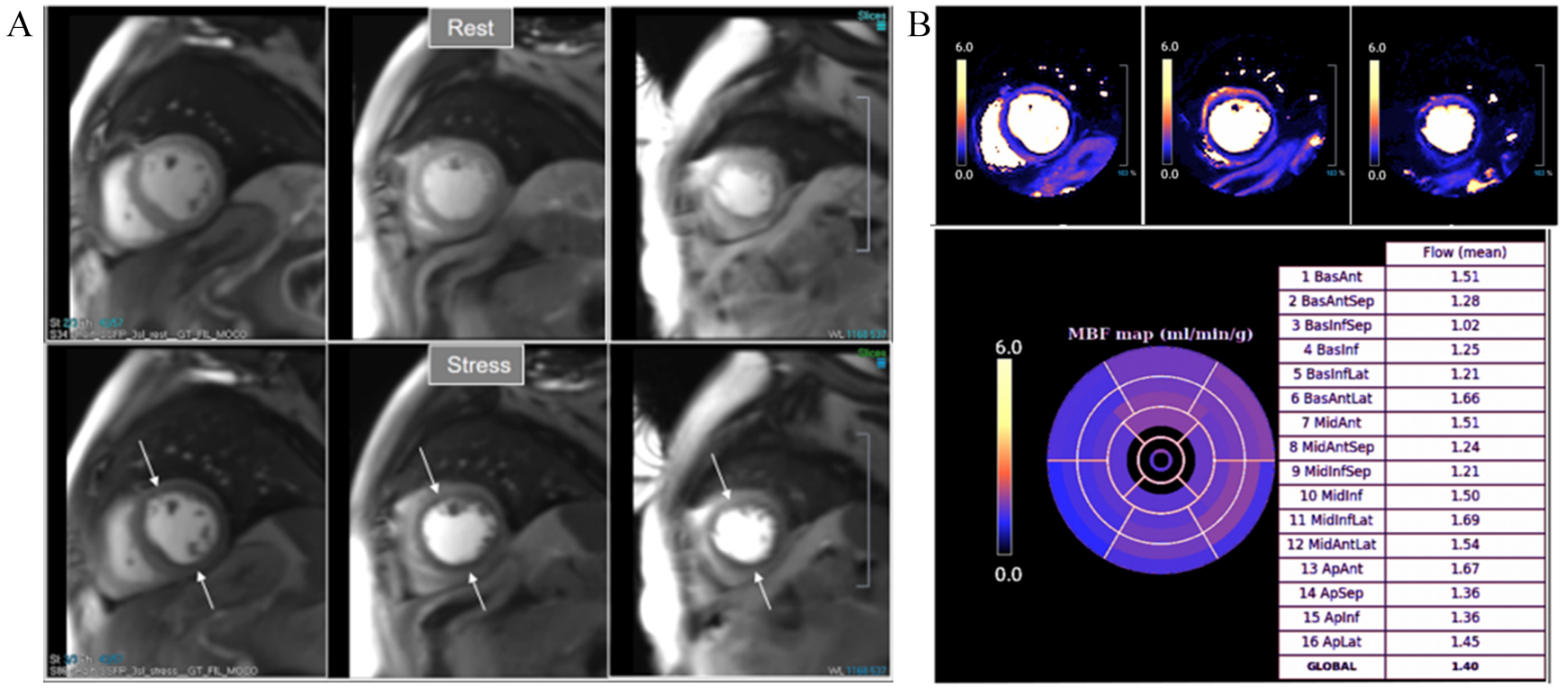
**Severely abnormal perfusion mapping**. (A) Rest and stress perfusion studies in a patient with severe advanced multivessel CAD showing a global subendocardial and partially transmural inducible defect (arrows). (B) Perfusion mapping showing extremely reduced values of stress MBF indicating multivessel obstructive disease.

Although introduced more recently, absolute QP using the dual-sequence approach [56] has been the subject of intense clinical investigation, starting with a comparison study with PET [79], still the recognized reference in myocardial blood flow quantitation, with which CMR showed an excellent correlation. Feasibility and good reproducibility of studies were established [80], the automatic in-line processing of data emerging as a unique tool removing the need for complex and time-consuming analysis of previous methods. A study comparing QP with quantitative coronary angiography found excellent diagnostic performance of the technique using thresholds of regional stress MBF of 1.29 mL/min/g and MPR of 1.47 for the detection of significant obstructive lesions (≥70%) [81]. With the reference of 3D reconstructed quantitative coronary angiography, an optimal diagnostic threshold of 1.5 mL/min/g of regional stress MBF was found, again for the detection of ≥70% coronary stenoses [82]. A key investigation where QP was performed in patients in whom invasive measures of coronary physiology (FFR and IMR) were available [83] established an optimal cut-off point for the detection of obstructive CAD (FFR <0.80) of 1.94 mL/min/g for regional stress MBF. Additionally, global stress MBF >2.25 mL/min/g was shown to be able to distinguish normal from abnormal coronary flow (either due to obstructive epicardial lesions or to MVD) and, to distinguish three-vessel disease from diffuse MVD, a cut-off point ≤1.82 mL/min/g was established. Advanced CAD frequently involves one or more epicardial obstructive lesions and MVD, either regional or global, and although cut-off values of stress MBF are progressively reduced, as observed, from normal perfusion to MVD and, then, to severe multi-vessel disease, overlapping is common in practice. Useful in this sense is to combine perfusion mapping data with visual assessment of defects into a diagnostic algorithm [83]: in brief, when a visual regional defect accompanies a matching reduced stress MBF (≤1.94 mL/min/g), obstructive epicardial coronary artery is the cause (Fig. 30); in the absence of a visible regional defect, a normal global stress MBF (>2.25 mL/min/g) would indicate normal coronary function (Fig. 32), while a global MBF below this value would suggest MVD (Fig. 33); finally, significant three-vessel disease should exhibit global stress MBF ≤1.82 mL/min/g and, importantly, a visual defect globally distributed (Fig. 34).

**Fig. 35.**
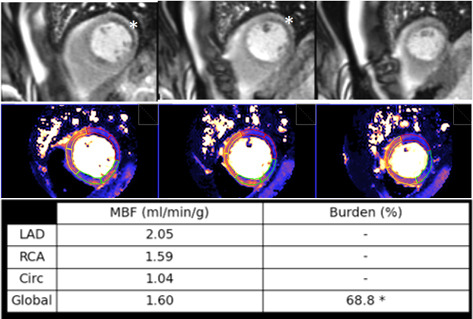
**Ischemic burden in multiple vessel disease as assessed by QP**. Stress perfusion (top row) showing a marked defect at the lateral wall (asterisks) while the assessment of the rest of segments is uncertain. Derived from the perfusion mapping (middle row), and assigned to coronary territories, are values of MBF (bottom row) indicating significant inducible ischemia for the territory of the circumflex artery, clear impairment of the RCA, and mostly preserved LAD region, all accounting for a global ischemic burden of 68.8%.

Multivessel disease is a scenario where the diagnostic performance of perfusion studies visually assessed is particularly challenging. Defects from different territories are not necessarily homogenous (Figs. 7,8,10), and CMR, although superior to SPECT, fails to identify visual defects in all territories in up to a third of patients with three-vessel disease [84]. This figure was shown to be as low as one-half in a subsequent study where QP was fairly more accurate (87%) than visual assessment for correctly identifying three-vessel disease when any coronary territory with ≥2 adjacent myocardial segments presenting with regional stress MBF <1.94 mL/min/g was considered as ischemic [85]. Based on this cut-off value [83, 85], an additional information derived from the dual sequence QP application is the calculation of a global ischemic burden with values of MBF assigned to every coronary territory (Fig. 35), which can be useful in the estimation of extension of the process.

MVD is another entity where the contribution of perfusion mapping has been essential, as the disorder is frequently overlooked due to the lack of appropriate, non-invasive, reliable tests for its assessment. While patients with MVD present with subendocardial hypoperfusion [41], a circumferential defective enhancement at first pass is detected at visual analysis of perfusion CMR only in a proportion of them (Fig. 9). A first study using QP CMR on patients with angina and no obstructive CAD showed that stress MBF and MPR were significantly reduced with respect to a control group of normal individuals [86]. A further study comparing QP CMR with invasive measurements of CFR confirmed these findings [87] establishing as optimal thresholds for the diagnosis a global MPR of 2.19 and, particularly, a subendocardial global MPR of 2.41. Interestingly, only 58% of patients with proven MVD presented with visually detectable defects in this study.

The prognostic value of perfusion mapping with automatic in-line flow measurement has been studied on a large population of 1049 patients with known or suspected CAD followed-up for a median of 605 days [88], showing that stress MBF and MPR are strong, independent predictors of adverse cardiovascular outcomes. Interestingly, MPR was superior to stress MBF in predicting death (although not a composite of events), an observation in accordance with a previous PET study [89] on a population of 4029 patients with a median follow-up of 5.6 years, where CFR (the equivalent of MPR) was a stronger predictor of cardiovascular mortality than maximal MBF. These findings seem to focus on the impaired vasodilator capacity, expressed by CFR or MPR, as a more relevant mechanism than peak MBF in estimating CAD risk.

In the subset of patients with previous CABG, a study of 341 patients with a mean follow-up of 1.7 years proved that both stress MBF and MPR independently predict adverse outcomes, with a cut-off point of 1.48 mL/min/g of mean stress MBF [90].

**Fig. 36.**
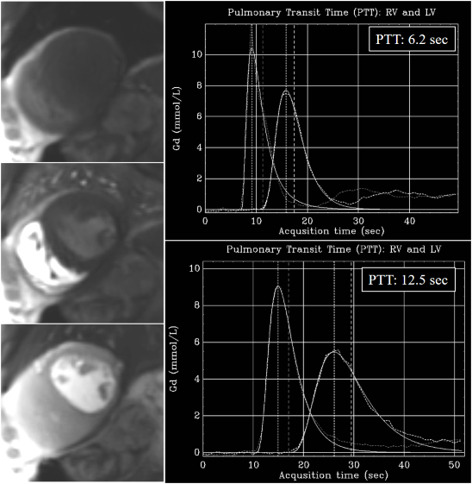
**PTT estimation from QP sequence**. Left panel: time frames from a dynamic rest perfusion sequence before the arrival of contrast to the heart (top), at the enhancement of the right ventricle (middle), and, subsequently, of the left one (bottom). Right panels: gadolinium time-concentration curves from which PTT is automatically calculated inline. Note the striking difference between values from a normal subject (6.2 sec) (top) and from a patient with advanced ischemic cardiomyopathy and ejection fraction of 21% (12.5 sec) (bottom).

Although unrelated with MBF, an additional parameter of interest that is automatically estimated in-line with the dual-sequence QP is the time interval for the contrast-bolus to pass from the right to the left circulation, or pulmonary transit time (PTT) (Fig. 36). As a measure of global cardiopulmonary function, PTT has shown to be independently associated with cardiovascular events [91], with longer values (>8 sec) observed in patients with higher incidence of events. PTT can thus be considered as a biomarker with prognostic information that is available in every study of QP without user interaction.

## 7. Position of Perfusion CMR in Current Clinical Practice

Attempting the relief of ischemia by revascularization, either PCI or coronary artery by-pass grafting (CABG), seems to be a matter of straightforward common sense and, in fact, it turned into a paradigm in clinical cardiology in the past. Nevertheless, the role of inducible ischemia as a target for a prompt intervention has been challenged thereafter by results from several large, randomized trials failing to show an improved long-term outcome of patients with CAD and objective evidence of myocardial ischemia when treated by PCI compared with medical management [92], even when the presence of more than mild ischemia had been proven on stress testing [93]. In contrast, several other studies of equally high level have shown that choosing to intervene only on vessels with definite flow obstruction by invasive FFR leads to a lower rate of events [94], and that this benefit persists over time [95].

Far from underestimate the value of ischemia, these disparities can also be interpreted as indicating a need for a refinement in its grading, an aspect where CMR perfusion methods discussed here could play a potential role. Of note, some of them, as QP, were not even available at the time these important studies were carried out.

In current practice, however, we have to refer to the present position of CMR perfusion in scientific guidelines for the management of patients with CAD. To be considered, first of all, is the fact that CMR is not treated particularly in most of them, but is included into the group of “non-invasive functional imaging”, together with SPECT, PET, and stress ECHO. Certainly, the usefulness of each of these techniques has been extensively proven, although the study of their relative value has shown a somewhat higher diagnostic accuracy of PET and CMR in comparison with SPECT and ECHO [96]. Logically enough, local expertise and availability of tests also should play a role in the selection.

This considered, non-invasive imaging merits a Class IB indication for initial diagnosis of CAD in symptomatic patients in whom obstructive CAD cannot be excluded by clinical assessment alone. Either, functional or anatomic (CCT) tests are valid options, their selection depending on the pre-test likelihood of the disease [4].

The precise term “myocardial ischemia” is referred to in the guidelines where a consideration of non-invasive tests is recognized (Class IIB) for its assessment, together with myocardial viability, in patients with CAD who are considered suitable for coronary revascularization [97] and, more straightforward, where a large area of ischemia by functional testing is considered as Class IB for prognostic purposes in revascularization planning [98]. From this last source, a list of “ten commandments” is issued [99] where two of them are particularly relevant in regard to myocardial ischemia: (1) “Objective evidence of myocardial ischemia by non-invasive stress imaging and/or intravascular assessment of the functional relevance of coronary artery stenoses are needed to indicate myocardial revascularization through PCI or CABG and to select the appropriate targets for PCI”; and (2) “With large areas of inducible myocardial ischemia or relevant LV systolic dysfunction myocardial revascularization through CABG or PCI is indicated to improve long-term survival”.

Importantly, a first direct reference to QP CMR appears in the most recent version of the American guidelines for management of chest pain [100], where stress CMR with the addition of MPR measurement is recognized as reasonable (Class IIA) for patients with persistent stable chest pain and nonobstructive CAD to improve diagnosis of coronary myocardial dysfunction and for estimating risk of events.

## 8. Perspectives for the Near Future of Recent Advances in CMR Perfusion

Conventional CMR is, thus, well positioned, among the diagnostic methods of ischemia. Advances in this area mainly relate to whole heart acquisition using 3T MR systems [101], with the derived increase in spatial coverage of perfusion studies.

Moreover, the availability of a reliable method for QP offers a wide range of new perspectives that will arguably lead to conceptual changes on the issue and, eventually, also into the adoption of new paradigms. Automatic workflow with pixelwise myocardial perfusion maps has immediate advantages in terms of reproducibility and objectivity avoiding the recognized drawback of visual perfusion analysis based on the heterogeneity of SI among different myocardial regions within the same study plane, where those taken as a reference are not invariably perfused normally. This will probably help to the recognition of “new” or insufficiently known aspects of CAD: subclinical, MVD, or relative regional ischemic burden in multivessel disease, all of them with a potential impact on the whole spectrum of IHD that deserves to be studied, and that may help to explain why the disease frequently ignites and escalates in an unpredictable way. Important, in this sense, is the potential of hybrid PET-MRI systems in combining molecular and structural imaging [102], where the information from PET on myocardial and, particularly, coronary artery inflammation [103], can be added to the rest of data from CMR imaging.

Also, and most important, a great change is coming for the CMR practitioner with the routine use of automatic QP applications, where a reliable set of automatically obtained numerical data must be integrated with the conventional reading of perfusion imaging [104]. Similar processes of automation have been also extended to other measurements of CMR [105], and a near future of studies fully based on outputs obtained from machine learning can be anticipated. The integration of this information requires both, a recognition of the limitations of our subjective analysis and a critical, well-informed evaluation of quantitative data. When considered into the clinical context of every patient, this new mode of information will prove beneficial in terms of diagnosis, prognosis, and therapy planning in IHD.

## 9. Conclusions

In conclusion, the assessment of myocardial perfusion is an essential part of a CMR study in patients with proven or suspected coronary artery disease. It has shown fairly good accuracy for the detection of the disease, compares favorably with other methods, and is useful for the prognostic stratification of patients. The recent introduction of a reliable technique of quantitative analysis with automatic, user-independent, processes of calculation of myocardial perfusion (in mL/min/g), myocardial segmentation, and allocation of values, constitutes a major step forward toward the consideration of CMR Perfusion as a unique diagnostic tool in the study of patients with ischemic heart disease.
